# Residual Positive Buttress Displacement After Femoral Neck System Fixation for Acute Femoral Neck Fractures: A Retrospective Cohort Study

**DOI:** 10.1111/os.70450

**Published:** 2026-10-22

**Authors:** Jian Xing, Yanan Ren, Pengfei Wang, Kun Zhang

**Affiliations:** ^1^ General Division of Orthopedic Trauma Department Honghui Hospital, Xi'an Jiaotong University Xi'an China

**Keywords:** femoral neck fracture, femoral neck system, Harris Hip Score, positive‐buttress reduction, reduction quality

## Abstract

**Objective:**

When anatomical reduction cannot be safely achieved, the functional relevance of residual positive‐buttress displacement after FNS fixation remains uncertain. Few clinical studies have quantified how its magnitude relates to hip function. To determine whether hip function after femoral neck system fixation differs by postoperative reduction pattern and, within positive‐buttress reduction, by residual medial cortical displacement.

**Methods:**

This retrospective cohort study included 180 adults with isolated acute femoral neck fractures treated with femoral neck system fixation between September 1, 2023, and April 30, 2024, and followed for at least 12 months. The overall analysis included Garden I–IV fractures; the exploratory five‐group displacement analysis was restricted to Garden I–III fractures. Postoperative reduction was classified as anatomical reduction, positive‐buttress reduction, or negative‐buttress reduction. Positive‐buttress displacement was measured on anteroposterior radiographs and categorized as PR‐2 (≤ 2 mm), PR‐4 (> 2–4 mm), or PR‐6 (> 4 mm). The primary outcome was 12‐month Harris Hip Score. Group comparisons used one‐way analysis of variance, Kruskal–Wallis tests, chi‐square or exact tests, and multivariable linear regression. Multivariable linear regression adjusted for age, sex, and Garden classification. Additional analyses evaluated continuous displacement, retained Garden IV fractures in the five‐group comparison, and incorporated the available clinical covariates.

**Results:**

The cohort included 45 anatomical, 98 positive‐buttress, and 37 negative‐buttress reductions. Median 12‐month Harris Hip Score was 95.8, 86.0, and 69.5, respectively. Compared with anatomical reduction, adjusted Harris Hip Score was lower by 9.40 points in positive‐buttress reduction and 25.62 points in negative‐buttress reduction. In the exploratory analysis restricted to Garden I‐III fractures, median Harris Hip Score declined across anatomical reduction, PR‐2, PR‐4, PR‐6, and negative‐buttress reduction (95.8, 90.0, 80.0, 74.0, and 68.9). Among the 82 positive‐buttress cases in this restricted analysis, each additional millimeter of displacement was associated with a 3.89‐point lower score (95% confidence interval, 3.12–4.66 points lower). The additional five‐group analysis retaining Garden IV fractures showed the same direction of association.

**Conclusions:**

In the overall cohort, reduction pattern was associated with 12‐month hip function. Garden IV fractures and open reduction were unevenly distributed across reduction patterns, and adjustment cannot eliminate residual confounding from fracture complexity, reduction difficulty, surgeon decisions, and unmeasured factors; these associations do not show that changing reduction pattern improves function. In the exploratory analysis restricted to Garden I‐III fractures, PR‐2 had the highest 12‐month Harris Hip Score among the nonanatomical groups. Larger residual displacement was associated with lower scores, but the findings do not establish a clinical 2‐mm threshold or a clinically important PR‐2 versus anatomical reduction difference. The additional all‐Garden analysis does not constitute separate validation in Garden IV fractures. PR‐2, PR‐4, and PR‐6 were exploratory categories rather than validated clinical thresholds or indications for additional reduction or open surgery.

## Graphical abstract

In adults treated with femoral neck system fixation, hip function declined as residual medial cortical displacement increased. Small positive‐buttress offset (≤ 2 mm) was the least unfavorable nonanatomical pattern, whereas larger offset and negative‐buttress reduction were linked to worse 12‐month Harris Hip Scores.

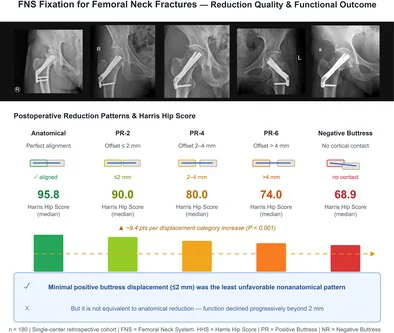

## 1 Introduction

Adult femoral neck fractures remain challenging because head‐preserving treatment must achieve union while preserving femoral head viability, femoral neck length, and hip function. Reoperation, avascular necrosis, femoral neck shortening, and persistent functional limitation remain clinically important after internal fixation [1, 2, 3, 4, 5].

Reduction quality is among the few modifiable intraoperative determinants of outcome. Anatomical reduction (AR) is preferred, but repeated manipulation, forceful reduction, or open reduction may add surgical trauma or compromise local biology. Fracture verticality, posterior tilt, surgical delay, and postoperative loading also influence fixation failure and function [5, 6, 7, 8, 9]. The practical question is therefore what residual deformity can be accepted when perfect reduction cannot be safely attained.

Gotfried positive‐buttress reduction (PR) reframed non‐AR as potentially acceptable in selected cases [10, 11, 12]. Clinical and biomechanical studies suggest that PR may be more favorable than negative‐buttress reduction (NR) because inferomedial cortical support is at least partly preserved [11, 12, 13, 14, 15].

However, previous studies have generally treated PR as a single category. This limits interpretation of residual displacement after FNS fixation when AR cannot be safely achieved. Minimal medial cortical offset is unlikely to have the same mechanical or functional implications as larger displacement, particularly with the femoral neck system (FNS), which provides angular and sliding stability and has been evaluated in biomechanical, prospective, and retrospective clinical studies [16, 17, 18, 19, 20, 21, 22, 23, 24, 25, 26] However, FNS cannot compensate for all consequences of suboptimal reduction morphology. We therefore addressed three questions: (i) whether postoperative reduction pattern was associated with 12‐month HHS; (ii) whether HHS varied across exploratory PR‐2, PR‐4, and PR‐6 categories; and (iii) how Garden classification, open reduction, reduction pattern, and recorded other factors affected hip function. We hypothesized that NR would be associated with the least favorable outcomes and that, within PR, 12‐month HHS would be lower as displacement increased.

## 2 Methods

### 2.1 Study Design and Setting

This retrospective cohort study was approved by the Ethics Committee of Honghui Hospital, Xi'an Jiaotong University (approval no. 202511043) and was reported in accordance with the STROBE statement. The informed consent was waived because of the retrospective design. Adults were defined as patients aged 18 years or older, with no upper‐age eligibility limit. Patients with isolated acute femoral neck fractures treated with FNS fixation between September 1, 2023, and April 30, 2024, were reviewed. The FNS is indicated for femoral neck fractures (AO/OTA 31‐B). Garden classification was based on preoperative anteroposterior radiographs: Type I, incomplete or valgus‐impacted; Type II, complete and nondisplaced; Type III, partially displaced; and Type IV, fully displaced. Open reduction was defined by an operative record documenting direct exposure of the femoral neck fracture site. The choice of fixation or arthroplasty was made by the treating surgeons in routine practice, taking patient age and fracture displacement into account; FNS was selected when the treating team judged head‐preserving internal fixation appropriate. FNS treatment was not assigned by this retrospective study. Eligible patients had a minimum follow‐up of 12 months. Follow‐up was terminated early if a patient underwent reoperation or died. HHS assessments were scheduled at approximately 6, 12, and 18 months. Recovery of preinjury work capacity was assessed at each patient's last available follow‐up, rather than at a uniform 18‐month visit.

The study was conducted across seven orthopedic trauma wards at a single tertiary trauma center. All patients underwent internal fixation with a single FNS (DePuy Synthes, Zuchwil, Switzerland) device according to standard institutional practice. The standard FNS antirotation screw was used in every patient; no additional screw was inserted. Patients were positioned supine on a fracture table, and reduction and implant placement were performed under fluoroscopic guidance. Postoperative rehabilitation followed a protected staged pathway, beginning with protected ambulation using bilateral crutches and progressing to single‐crutch support and full weight bearing. Treating physicians individualized progression at follow‐up according to pain, clinical function, and radiographic healing; there was no identical fixed schedule for all patients or reduction groups. Rehabilitation records were expressed in weeks. Bilateral‐crutch use was recorded as a duration. The remaining recorded rehabilitation fields and their between‐group comparisons are presented in Tables 1 and S6.

**TABLE 1:** Baseline and perioperative characteristics of the overall cohort by postoperative reduction pattern.

	AR (*n* = 45)	PR (*n* = 98)	NR (*n* = 37)	*p*
Age, years (mean ± SD)	41.6 ± 12.2	42.5 ± 15.7	38.9 ± 11.8	0.414
Female sex, *n* (%)	27 (60.0%)	42 (42.9%)	17 (45.9%)	0.158
Follow‐up, medians, ranges (weeks)	18.7 (15.4–21.6)	18.4 (15.1–19.7)	18.2 (17.8–21.4)	—
Follow‐up at 6, 12, and 18 months, *n*/*N* (%)	45/45 (100.0%); 45/45 (100.0%); 42/45 (93.3%)	98/98 (100.0%); 98/98 (100.0%); 93/98 (94.9%)	37/37 (100.0%); 37/37 (100.0%); 36/37 (97.3%)	—
Garden classification				< 0.001
Garden I, *n* (%)	5 (11.1%)	3 (3.1%)	5 (13.5%)	
Garden II, *n* (%)	18 (40.0%)	33 (33.7%)	5 (13.5%)	
Garden III, *n* (%)	15 (33.3%)	46 (46.9%)	10 (27.0%)	
Garden IV, *n* (%)	7 (15.6%)	16 (16.3%)	17 (45.9%)	
Open reduction, *n* (%)	4 (8.9%)	13 (13.3%)	12 (32.4%)	0.012
Injury‐to‐surgery interval, days, median (range)	3.8 (0.3–15.4)	3.4 (0.2–11.9)	2.8 (0.2–10.8)	0.549
Pauwels angle, degrees, median (range)	53.8 (34.1–71.4)	56.9 (29.6–83.5)	60.0 (31.2–81.7)	0.123
Posterior tilt angle, degrees, median (range)	11.3 (0.0–14.7)	9.9 (0.2–13.4)	10.4 (0.0–12.5)	0.670
Body mass index (BMI), kg/m^2^, median (range)	23.8 (16.5–31.6)	23.9 (16.5–31.8)	23.1 (16.5–31.9)	0.611
Smoking status, *n* (%)	Never 8 (17.8%); Former 4 (8.9%); Current 33 (73.3%)	Never 31 (31.6%); Former 8 (8.2%); Current 59 (60.2%)	Never 9 (24.3%); Former 4 (10.8%); Current 24 (64.9%)	0.512
FNS standard antirotation screw, *n* (%)	45 (100.0%)	98 (100.0%)	37 (100.0%)	—
Additional screw, *n* (%)	0 (0.0%)	0 (0.0%)	0 (0.0%)	—
Implant tip‐apex distance, mm, median (range)	20.3 (9.0–28.3)	20.9 (9.0–33.4)	20.5 (12.3–28.9)	0.384
Implant position category, *n* (%)	Central 41 (91.1%); Eccentric 4 (8.9%)	Central 79 (80.6%); Eccentric 19 (19.4%)	Central 28 (75.7%); Eccentric 9 (24.3%)	0.158
Non‐weightbearing record, weeks, mean ± SD	4.91 ± 0.57	5.10 ± 0.97	5.21 ± 0.50	0.214
Bilateral‐crutch duration, weeks, mean ± SD	1.22 ± 0.11	1.30 ± 0.19	1.80 ± 0.10	< 0.001
Single‐crutch record, weeks, mean ± SD	3.11 ± 0.57	3.25 ± 0.52	2.91 ± 0.50	0.016
Full‐weightbearing record, weeks, mean ± SD	10.32 ± 1.26	9.76 ± 1.61	9.40 ± 1.00	0.021
Primary surgeons, *n* (case‐count range)	18 (1–6)	22 (1–8)	16 (1–8)	—
Patient‐level ward assignment	Unavailable	Unavailable	Unavailable	—

*Note:* Age and rehabilitation fields are mean ± SD; other continuous variables are median (range). Rehabilitation fields are in weeks.

Abbreviations: AR, anatomical reduction; NR, negative‐buttress reduction; PR, positive‐buttress reduction; SD, standard deviation.

### 2.2 Patient Selection

The derivation of the final analytic cohort is summarized in Figure 1. Eligible participants were adults (≥ 18 years) with isolated acute femoral neck fractures treated with FNS fixation within 30 days of injury, with complete data for the primary analysis and standard postoperative anteroposterior and lateral radiographs. Exclusion criteria were: (1) concomitant fractures at other anatomical sites; (2) treatment other than internal fixation, including arthroplasty or nonoperative treatment; (3) surgery performed more than 30 days after injury; (4) pathological, nontraumatic fractures; (5) incomplete clinical or imaging data; (6) inability to ambulate on the affected limb or to perform usual work or household duties before injury; (7) preexisting deformity or claudication of the affected limb; (8) prior surgery on the affected limb; (9) preoperative avascular necrosis (AVN) of the femoral head; (10) loss to follow‐up; and (11) inadequate postoperative imaging, defined as absence of standard anteroposterior and lateral radiographs, or head–neck angular displacement greater than 15° on the lateral view.

**FIGURE 1:**
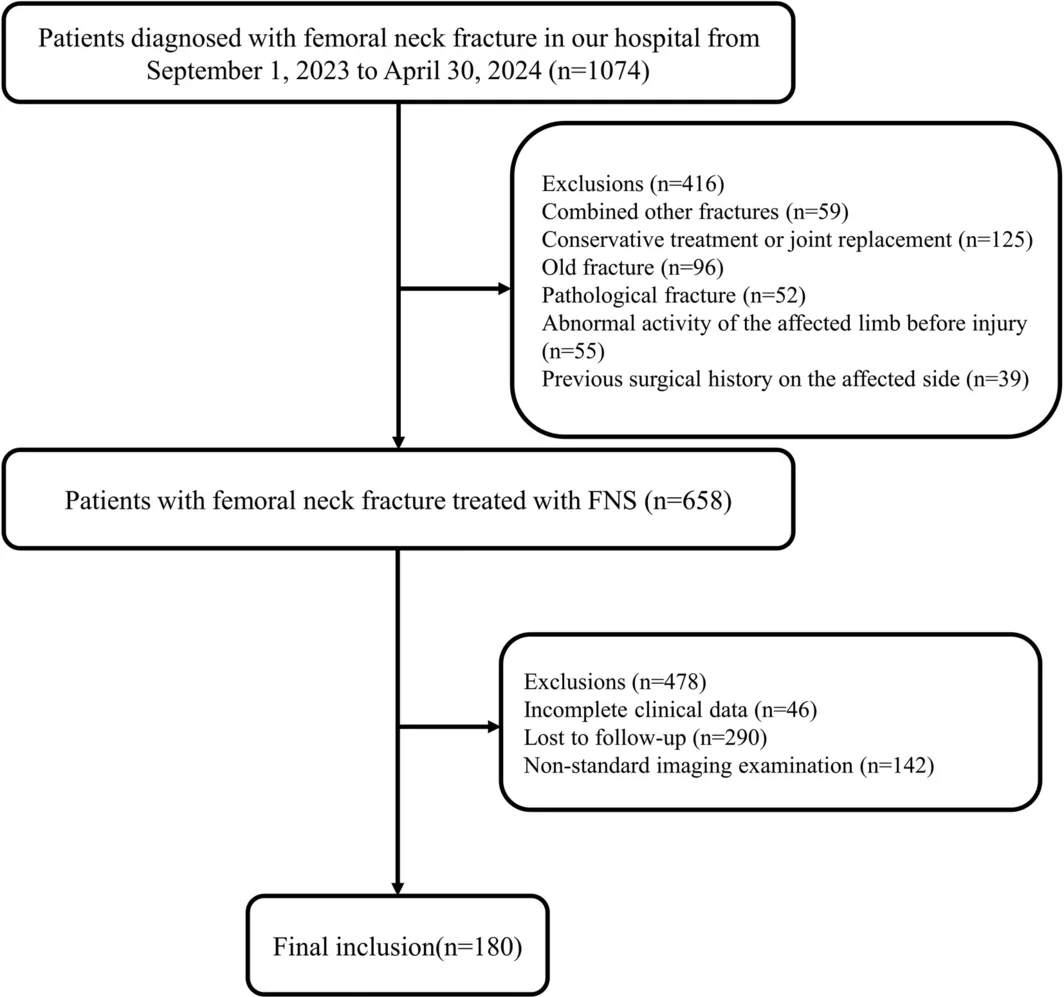
Patient selection flowchart. Flowchart showing identification of adult patients with acute femoral neck fractures treated with femoral neck system fixation and derivation of the final analytic cohort. FNS, femoral neck system.

### 2.3 Outcomes and Comparisons

Postoperative follow‐up data were collected from clinical and radiographic assessments. Hip function was evaluated using the Harris Hip Score (HHS) at approximately 6, 12, and 18 months after surgery. The primary outcome was 12‐month HHS because it was available for all patients and was less influenced by early postoperative rehabilitation restrictions than the 6‐month assessment. HHS at 6 and 18 months was analyzed as secondary time point‐specific functional outcomes. Complications recorded during follow‐up included secondary surgery, AVN of the femoral head, and femoral neck shortening. Secondary surgery was defined as any unplanned reoperation related to the index fracture or fixation construct. AVN was diagnosed according to serial imaging findings and clinical records. Femoral neck shortening was assessed on follow‐up radiographs when adequate imaging was available. Recovery of preinjury work capacity was defined as the ability to resume paid work or principal unpaid household work performed before injury. It represented recovery of the patient's preinjury productive role, rather than paid employment alone. All included patients had this capacity before injury; patients with preinjury limitations precluding their usual work or household role were not eligible. The outcome was assessed at the last available follow‐up. The numerator was the number able to resume their preinjury role, and the denominator was all patients in the relevant analysis group. Because event numbers were expected to be small, these secondary outcomes were interpreted descriptively.

HHS assessors were blinded to postoperative reduction patterns. Separately, the radiographic evaluators were blinded to HHS and complication outcomes. This masking reduced the risk of assessment bias related to knowledge of the reduction pattern.

Reduction quality was assessed on the first standard postoperative anteroposterior radiograph. Three independent evaluators reviewed all radiographs and classified reduction as AR, PR, or NR according to the relative position of the medial cortices at the fracture site. Evaluators were blinded to postoperative HHS and complication outcomes during image review.

AR was defined as the absence of a discernible cortical step on orthogonal anteroposterior and lateral radiographs. The common lateral‐view eligibility criterion remained as described below.

For cases classified as PR, the relative displacement between the medial cortices was independently measured by the three evaluators on the first postoperative anteroposterior radiograph. Measurements were performed in the picture archiving and communication system after calibration to the known FNS bolt diameter of 10 mm to reduce magnification‐related error. The measured distance was between the projected medial cortical edges at the fracture line on the calibrated anteroposterior image (Figure 2). The mean of the three measurements was used as the final displacement value for subgroup classification. PR cases were stratified into PR‐2 (≤ 2 mm), PR‐4 (> 2–4 mm), and PR‐6 (> 4 mm). The 2‐ and 4‐mm boundaries were informed by the finite element study of Wang et al., which examined 2‐, 3‐, and 4‐mm positive offsets in a cannulated‐screw Pauwels I model [14]. We used these boundaries as exploratory categories of smaller, intermediate, and larger residual displacement, not as validated clinical thresholds or indications for additional reduction or open surgery. Continuous‐displacement and quadratic sensitivity analyses were also performed (Table S1). PR‐6 denotes the > 4‐mm category.

**FIGURE 2:**
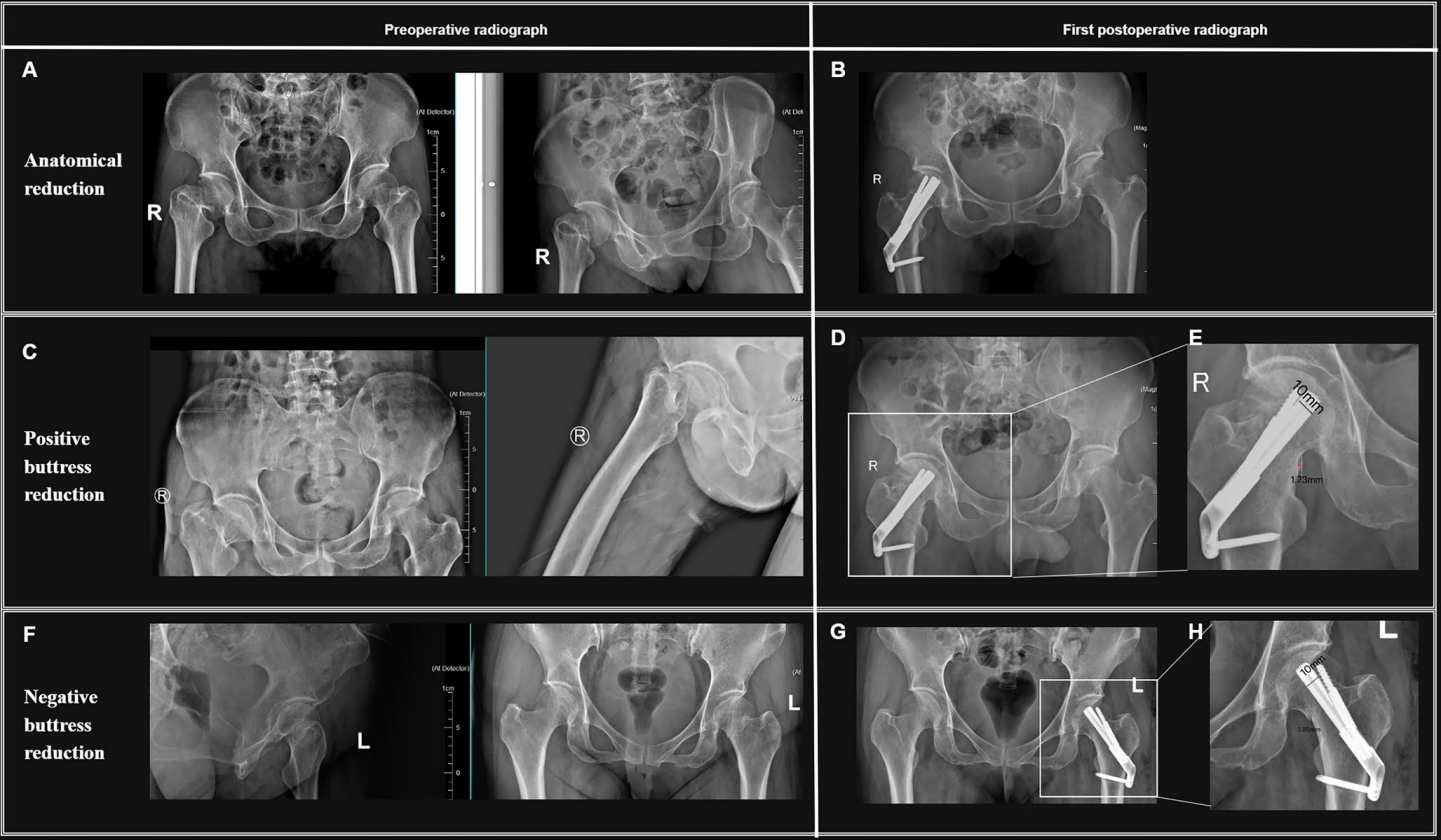
Representative reduction patterns and cortical displacement measurements after femoral neck system fixation. The first standard postoperative anteroposterior radiograph was calibrated using the known 10‐mm diameter of the FNS bolt. Residual positive‐buttress displacement was measured between the projected medial cortical edges at the fracture line. The final displacement value was the mean of three independent evaluator measurements and was used to classify PR‐2 (≤ 2 mm), PR‐4 (> 2–4 mm), or PR‐6 (> 4 mm). FNS, femoral neck system; PR, positive‐buttress reduction.

To focus the displacement analysis on coronal‐plane medial cortical offset rather than combined multiplanar deformity, lateral radiographs were reviewed concurrently. Patients were included only when anterior or posterior angulation on the lateral view did not exceed 15° and continuity of at least one anterior or posterior cortex was preserved.

The primary comparison evaluated postoperative function across AR, PR, and NR in the overall cohort including Garden I–IV fractures, using AR as the reference group. An exploratory five‐group analysis was restricted to Garden I–III fractures to examine the association in a less severely displaced case mix, given the unequal distribution of Garden IV fractures across reduction groups. This restriction changes the target population and does not eliminate residual confounding. In this restricted analysis, AR served as the reference group for comparisons with PR‐2, PR‐4, PR‐6, and NR. A separate trend analysis was performed across PR‐2, PR‐4, and PR‐6 using ordinal displacement categories. To address the clinically important Garden IV subgroup, an additional five‐group sensitivity analysis retained all 180 patients, including all 40 Garden IV fractures, and adjusted for Garden classification as a categorical variable.

### 2.4 Reliability Assessment

Interobserver agreement for categorical classification of reduction pattern (AR, PR, and NR) was assessed across all included patients using Fleiss kappa. Reliability of continuous displacement measurements was evaluated among patients with PR using intraclass correlation coefficients derived from a two‐way random‐effects model with absolute agreement. Both the single‐measure intraclass correlation coefficient [ICC(2,1)] and the average‐measure intraclass correlation coefficient [ICC(2,3)] were calculated. Confidence intervals for Fleiss kappa and intraclass correlation coefficient estimates were obtained by bootstrap resampling.

### 2.5 Statistical Analysis

Continuous variables are presented as mean ± standard deviation or median (range), as appropriate, and categorical variables as counts and percentages. Age was compared by one‐way analysis of variance, and recorded perioperative continuous variables by the Kruskal–Wallis test. Unadjusted HHS comparisons at each scheduled visit also used the tie‐corrected Kruskal–Wallis test, with available cases at 18 months and no multiplicity correction for these exploratory comparisons. Categorical comparisons used the chi‐square test or an exact test, as appropriate. Open‐reduction and binary secondary‐outcome comparisons used two‐sided Fisher–Freeman–Halton exact tests; the PR ≤ 2‐mm versus > 2‐mm comparison used Fisher's exact test with a conditional odds ratio and exact 95% confidence interval (CI). The association between reduction pattern and 12‐month HHS was estimated by multivariable linear regression adjusted for age, sex, and categorical Garden classification. Conventional model CIs used the *t* distribution.

Model 1 was the base model adjusted for continuous age, sex, and categorical Garden classification. Model 2 was the expanded clinical model. Expanded models additionally included open reduction, injury‐to‐surgery interval, Pauwels angle, posterior tilt angle, body mass index (BMI), and smoking status. Both models are presented for the primary overall three‐group comparison (Table 2) and the exploratory Garden I–III five‐group comparison (Table 3). The original Model 1 results retain conventional *t*‐based confidence intervals; Model 2 uses HC3 *t*‐based inference. Corresponding HC3 base models and the all‐Garden five‐group sensitivity models are reported in the supplement. Population restrictions and covariate adjustment define separate analysis dimensions.

**TABLE 2:** Association between postoperative reduction pattern and 12‐month Harris Hip Score in the overall cohort (*n* = 180) base and expanded models.

Comparison	Model 1 HHS difference (95% CI)	*p*	Model 2 HHS difference (95% CI)	*p*
PR vs. AR	−9.40 (−11.69 to −7.11)	< 0.001	−9.55 (−11.54 to −7.56)	< 0.001
NR vs. AR	−25.62 (−28.51 to −22.73)	< 0.001	−25.36 (−27.32 to −23.40)	< 0.001

*Note:* Negative estimates indicate lower HHS than AR. Model 1 adjusts for continuous age, sex, and categorical Garden classification and retains the original conventional *t*‐based inference. Model 2 additionally adjusts for open reduction, injury‐to‐surgery interval, Pauwels angle, posterior tilt, BMI, and smoking, using HC3 *t*‐based inference. Both models use the same patients. *p*‐Values are two‐sided and exploratory, without multiplicity adjustment. The corresponding all‐HC3 age‐unrestricted versus age‐ < 60 comparisons are reported in Table S12.

Abbreviations: AR, anatomical reduction; CI, confidence interval; HHS, Harris Hip Score; NR, negative‐buttress reduction; PR, positive‐buttress reduction.

**TABLE 3:** Exploratory association between postoperative reduction pattern and 12‐month Harris Hip Score after excluding Garden IV fractures (*n* = 140) base and expanded models.

Comparison	Model 1 HHS difference (95% CI)	*p*	Model 2 HHS difference (95% CI)	*p*
PR‐2 vs. AR	−4.54 (−6.65 to −2.43)	< 0.001	−4.36 (−6.29 to −2.44)	< 0.001
PR‐4 vs. AR	−14.25 (−16.47 to −12.04)	< 0.001	−14.18 (−16.60 to −11.77)	< 0.001
PR‐6 vs. AR	−22.60 (−25.84 to −19.35)	< 0.001	−22.29 (−26.73 to −17.85)	< 0.001
NR vs. AR	−24.92 (−27.48 to −22.37)	< 0.001	−24.73 (−27.17 to −22.29)	< 0.001

*Note:* Negative estimates indicate lower HHS than AR. Model 1 adjusts for continuous age, sex, and categorical Garden classification and retains the original conventional *t*‐based inference. Model 2 additionally adjusts for open reduction, injury‐to‐surgery interval, Pauwels angle, posterior tilt, BMI, and smoking, using HC3 t‐based inference. Both models use the same patients. *p*‐Values are two‐sided and exploratory, without multiplicity adjustment. The corresponding all‐HC3 age‐unrestricted versus age‐ < 60 comparisons are reported in Table S12. This five‐group analysis is restricted to Garden I–III fractures.

Abbreviations: AR, anatomical reduction; CI, confidence interval; HHS, Harris Hip Score; NR, negative‐buttress reduction; PR, positive‐buttress reduction.

The primary model used complete cases without imputation. Eighteen‐month HHS was analyzed using available cases. The primary models were refitted with heteroscedasticity‐consistent HC3 standard errors, using *t*‐based inference with residual degrees of freedom. Further sensitivity analyses used median quantile regression, restriction to Garden II–III fractures, an ordinal five‐level exposure after excluding Garden IV fractures, and generalized overlap weighting based on age, sex, and Garden classification. Continuous PR displacement was modeled per millimeter in all PR cases and separately in Garden I–III PR cases; a centered quadratic term assessed curvature without searching for a cutoff. Implant tip‐apex distance and position were added in a further sensitivity model. Standard FNS antirotation screws and absence of additional screws were invariant and could not be estimated as separate effects. Surgeon analyses used the 23 recorded surgeon codes, small‐sample‐corrected clustered standard errors, leave‐one‐surgeon‐out refitting, and surgeon fixed effects. Ward membership was not inferred from surgeon codes. Rehabilitation fields were entered one at a time as postoperative sensitivity covariates, rather than treated as preoperative confounders. Group differences in the four rehabilitation fields were assessed by Kruskal–Wallis tests with Holm correction, and their associations with 6‐month HHS, 12‐month HHS, and paired 6–12‐month HHS change by Spearman correlations with Holm correction within each set of 12 comparisons. Repeated HHS observations were modeled with group‐by‐time interactions and patient‐clustered standard errors. Complication occurrence dates were unavailable, so complication‐timing analyses were not performed. Model specifications, diagnostics, additional results, and interpretation limits are detailed in the Supplementary Statistical Analyses. All regression and open‐reduction analyses were exploratory; their *p* values were not corrected for multiplicity. A two‐sided *p* value < 0.05 was considered statistically significant.

An additional sensitivity analysis was restricted to patients younger than 60 years. Base and expanded models were refitted within the same three‐group, five‐group, and PR‐only populations, retaining continuous age as a covariate. Age‐unrestricted and age‐restricted estimates were compared using identical covariate sets and HC3 *t*‐based inference (Table S12).

## 3 Results

### 3.1 Study Population and Baseline Characteristics

A total of 180 adult patients with acute femoral neck fractures treated with FNS fixation were included in the final analysis (Figure 1). The mean age was 41.6 ± 14.1 years, and the cohort comprised 86 women and 94 men. According to the Garden classification, 13 fractures (7.2%) were Garden I, 56 (31.1%) were Garden II, 71 (39.4%) were Garden III, and 40 (22.2%) were Garden IV. All variables required for the primary model were complete. Among scheduled HHS assessments, 18‐month HHS was missing for nine of 180 patients (5.0%).

According to postoperative reduction pattern, 45 patients (25.0%) were classified as AR, 98 (54.4%) as PR, and 37 (20.6%) as NR. Sex distribution was broadly comparable across groups. The NR group contained 17/37 (45.9%) Garden IV fractures, compared with 7/45 (15.6%) in AR and 16/98 (16.3%) in PR (Table 1). This imbalance was addressed through covariate adjustment, an additional all‐Garden five‐group analysis, Garden II–III restriction, and overlap‐weighted analysis. Open reduction was performed in 4/45 AR patients (8.9%), 13/98 PR patients (13.3%), and 12/37 NR patients (32.4%; exact *p* = 0.012). The recorded rehabilitation fields had overall means of 5.07 ± 0.81 weeks for non‐weightbearing, 1.38 ± 0.27 weeks for bilateral‐crutch duration, 3.14 ± 0.54 weeks for single‐crutch use, and 9.83 ± 1.45 weeks for full weight bearing. These records do not by themselves establish postoperative initiation weeks. Tables 1 and S6 report the group‐specific means, standard deviations, and comparisons.

### 3.2 Reliability of Reduction Assessment

Across all 180 cases, Fleiss kappa for classification of AR, PR, and NR was 0.822 (bootstrap 95% CI, 0.759–0.880), indicating strong agreement among the three evaluators. Complete agreement among all three evaluators was observed in 152 of 180 cases (84.4%).

Among the 98 patients with PR, interobserver reliability for measurement of medial cortical displacement was also high. The single‐measure ICC was 0.896 (ICC[2,1]; bootstrap 95% CI, 0.864–0.918), and the average‐measure ICC for the mean of the three measurements used in the final analysis was 0.963 (ICC[2,3]; bootstrap 95% CI, 0.950–0.971).

### 3.3 Primary Functional Outcome in the Overall Cohort

Functional outcomes differed significantly among AR, PR, and NR (Table 4 and Figures 3 and 4). Individual HHS distributions over time are shown in Figure S1. Median HHS at 12 months, the primary endpoint, was 95.8 (82.2–100.0) in AR, 86.0 (64.0–100.0) in PR, and 69.5 (62.3–81.9) in NR. HHS at 18 months remained highest in AR (*n* = 42), intermediate in PR (*n* = 93), and lowest in NR (*n* = 36). At 6 months, median HHS was higher in NR (75.5) than in PR (73.7), so the ordering at this earlier visit differed from that at 12 months. The difference between the NR group medians at 6 and 12 months was −6.0 points; this is not an estimate of the median within‐patient change.

**TABLE 4:** Clinical outcomes of the overall cohort by postoperative reduction pattern.

	AR (*n* = 45)	PR (*n* = 98)	NR (*n* = 37)	*p*
Secondary surgery, *n* (%)	0 (0.0%)	0 (0.0%)	4 (10.8%)	0.002
Avascular necrosis, *n* (%)	0 (0.0%)	4 (4.1%)	3 (8.1%)	0.149
Femoral neck shortening, *n* (%)	1 (2.2%)	4 (4.1%)	4 (10.8%)	0.239
Recovery of preinjury work capacity, *n* (%)	8 (17.8%)	14 (14.3%)	3 (8.1%)	0.458
HHS at 6 months, median (range)	85.8 (72.2–96.3)	73.7 (54.6–91.5)	75.5 (68.3–87.9)	< 0.001
HHS at 12 months, median (range)	95.8 (82.2–100.0)	86.0 (64.0–100.0)	69.5 (62.3–81.9)	< 0.001
HHS at 18 months, median (range)	94.6 (84.5–100.0); *n* = 42	85.4 (63.5–100.0); *n* = 93	74.6 (67.3–86.9); *n* = 36	< 0.001

*Note:* Binary secondary‐outcome *p* values use a two‐sided.

Abbreviations: AR, anatomical reduction; HHS, Harris Hip Score; NR, negative‐buttress reduction; PR, positive‐buttress reduction.

**FIGURE 3:**
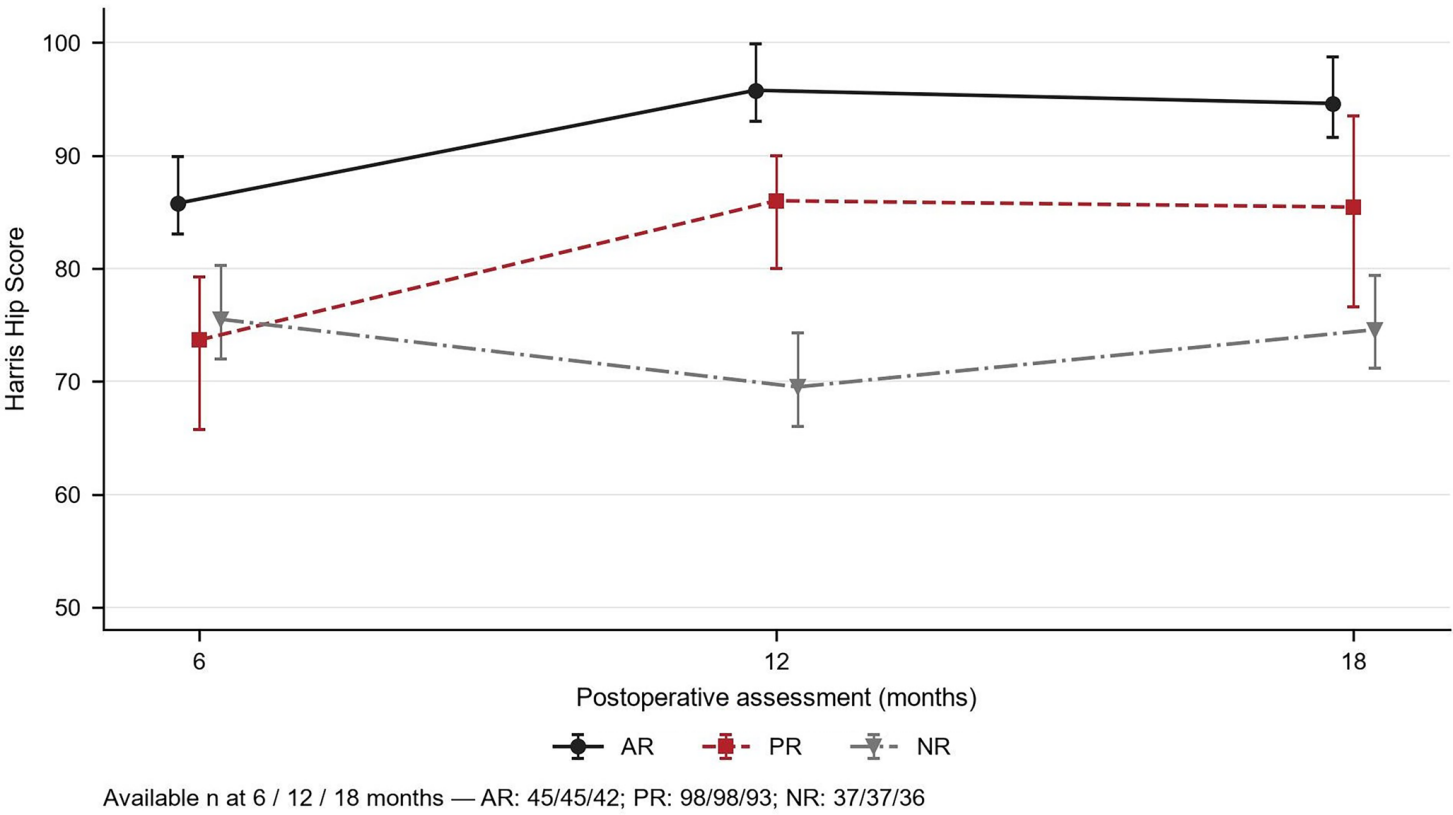
Longitudinal Harris Hip Score (HHS) summaries in the Garden I–IV cohort. Markers show the median and error bars the 25th–75th percentiles (interquartile range, IQR) at each recorded assessment. AR: 45, 45, 42; PR: 98, 98, 93; NR: 37, 37, 36 participants at 6, 12, and 18 months, respectively. AR, anatomical reduction; NR, negative‐buttress reduction; PR, positive‐buttress reduction. This is a descriptive figure; no significance test is encoded.

**FIGURE 4:**
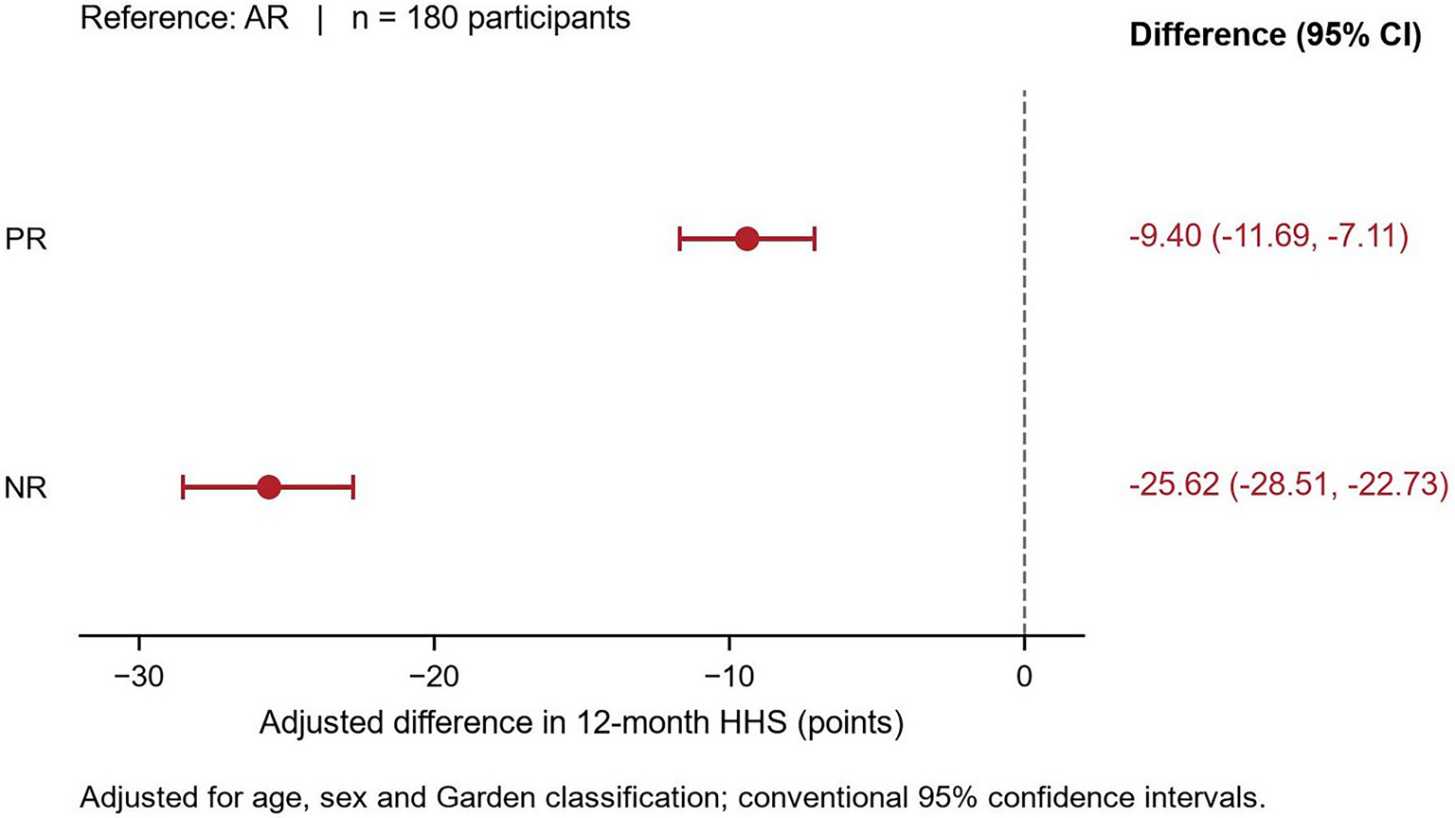
Adjusted between‐group differences in 12‐month Harris Hip Score (HHS) in 180 participants (Garden I–IV). Points are ordinary least‐squares regression coefficients relative to anatomical reduction (AR); horizontal bars are conventional two‐sided 95% confidence intervals, using Student's *t* distribution with residual degrees of freedom. Models adjust for age, sex, and categorical Garden classification. The dashed vertical line denotes no difference from AR. NR, negative‐buttress reduction; PR, positive‐buttress reduction. The plotted estimates correspond to Model 1 in Table 2.

In multivariable linear regression, both PR and NR were associated with lower 12‐month HHS than AR. Compared with AR, the adjusted difference in 12‐month HHS was −9.40 points (95% CI, −11.69 to −7.11) for PR and −25.62 points (95% CI, −28.51 to −22.73) for NR (Table 2). The expanded clinical estimates were −9.55 points for PR (95% CI, −11.54 to −7.56) and −25.36 for NR (−27.32 to −23.40); base and expanded results are shown together in Table 2.

### 3.4 Secondary Clinical Outcomes

Secondary surgery occurred only in the NR group (four of 37, 10.8%; *p* = 0.002). AVN was observed in 0 of 45 patients in the AR group, four of 98 in the PR group, and three of 37 in the NR group. Femoral neck shortening was documented in one patient in the AR group, four in the PR group, and four in the NR group. Recovery of preinjury work capacity at the last available follow‐up did not differ significantly among groups (Table 4).

### 3.5 Subgroup Analysis

After exclusion of Garden IV fractures, 140 patients remained for subgroup analysis: 38 in AR, 39 in PR‐2, 33 in PR‐4, 10 in PR‐6, and 20 in NR. Sex distribution and Garden I–III composition were generally comparable across these five groups; age summaries incorporate the seven confirmed entry corrections (Table 5).

**TABLE 5:** Baseline characteristics and clinical outcomes after excluding Garden IV fractures.

	AR (*n* = 38)	PR‐2 (*n* = 39)	PR‐4 (*n* = 33)	PR‐6 (*n* = 10)	NR (*n* = 20)	*p*
Age, years (mean ± SD)	41.6 ± 11.5	41.4 ± 16.5	42.7 ± 16.8	43.6 ± 17.9	39.3 ± 13.8	0.936
Female sex, *n* (%)	22 (57.9%)	18 (46.2%)	12 (36.4%)	5 (50.0%)	10 (50.0%)	0.496
Follow‐up, medians, ranges (weeks)	18.6 (17.8–20.2)	18.8 (16.3–19.8)	18.6 (17.0–20.3)	18.6 (17.8–20.6)	18.2 (17.8–20.0)	—
Follow‐up at 6, 12, and 18 months, *n*/*N* (%)	38/38 (100.0%); 38/38 (100.0%); 36/38 (94.7%)	39/39 (100.0%); 39/39 (100.0%); 35/39 (89.7%)	33/33 (100.0%); 33/33 (100.0%); 33/33 (100.0%)	10/10 (100.0%); 10/10 (100.0%); 10/10 (100.0%)	20/20 (100.0%); 20/20 (100.0%); 19/20 (95.0%)	—
Garden classification						0.135
Garden I, *n* (%)	5 (13.2%)	1 (2.6%)	1 (3.0%)	1 (10.0%)	5 (25.0%)	
Garden II, *n* (%)	18 (47.4%)	16 (41.0%)	13 (39.4%)	4 (40.0%)	5 (25.0%)	
Garden III, *n* (%)	15 (39.5%)	22 (56.4%)	19 (57.6%)	5 (50.0%)	10 (50.0%)	
Secondary surgery, *n* (%)	0 (0.0%)	0 (0.0%)	0 (0.0%)	0 (0.0%)	2 (10.0%)	0.024
Avascular necrosis, *n* (%)	0 (0.0%)	0 (0.0%)	1 (3.0%)	1 (10.0%)	1 (5.0%)	0.073
Femoral neck shortening, *n* (%)	1 (2.6%)	0 (0.0%)	1 (3.0%)	1 (10.0%)	2 (10.0%)	0.133
Recovery of preinjury work capacity, *n* (%)	3 (7.9%)	4 (10.3%)	2 (6.1%)	0 (0.0%)	1 (5.0%)	0.958
HHS at 6 months, median (range)	85.8 (72.2–96.2)	68.0 (54.7–91.5)	73.6 (54.6–89.4)	77.5 (60.4–88.1)	74.9 (69.4–87.9)	< 0.001
HHS at 12 months, median (range)	95.8 (82.2–100.0)	90.0 (80.0–100.0)	80.0 (69.0–92.0)	74.0 (64.0–78.0)	68.9 (63.4–81.9)	< 0.001
HHS at 18 months, median (range)	94.2 (84.5–100.0); *n* = 36	89.4 (77.1–100.0); *n* = 35	77.7 (63.5–89.6); *n* = 33	76.0 (64.0–83.4); *n* = 10	75.1 (68.4–86.9); *n* = 19	< 0.001
Open reduction, *n* (%)	2 (5.3%)	8 (20.5%)	3 (9.1%)	1 (10.0%)	4 (20.0%)	0.227
Additional reduction maneuvers, *n* (%)	Not recorded separately	Not recorded separately	Not recorded separately	Not recorded separately	Not recorded separately	—

Abbreviations: AR, anatomical reduction; HHS, Harris Hip Score; NR, negative‐buttress reduction; PR‐2, positive‐buttress displacement ≤ 2 mm; PR‐4, > 2–4 mm; PR‐6, > 4 mm; SD, standard deviation.

Median HHS at 12 months showed an ordered gradient across AR, PR‐2, PR‐4, PR‐6, and NR: 95.8 (82.2–100.0), 90.0 (80.0–100.0), 80.0 (69.0–92.0), 74.0 (64.0–78.0), and 68.9 (63.4–81.9), respectively (Table 5 and Figures 5 and 6). Individual HHS distributions for this subgroup analysis are shown in Figure S2. At 6 months, the corresponding medians were 85.8, 68.0, 73.6, 77.5, and 74.9. PR‐2 had the lowest median, whereas PR‐6 had the highest median among nonanatomical groups. Between 6 and 12 months, the PR‐2 group median increased by 22.0 points and the PR‐6 group median decreased by 3.5 points.

**FIGURE 5:**
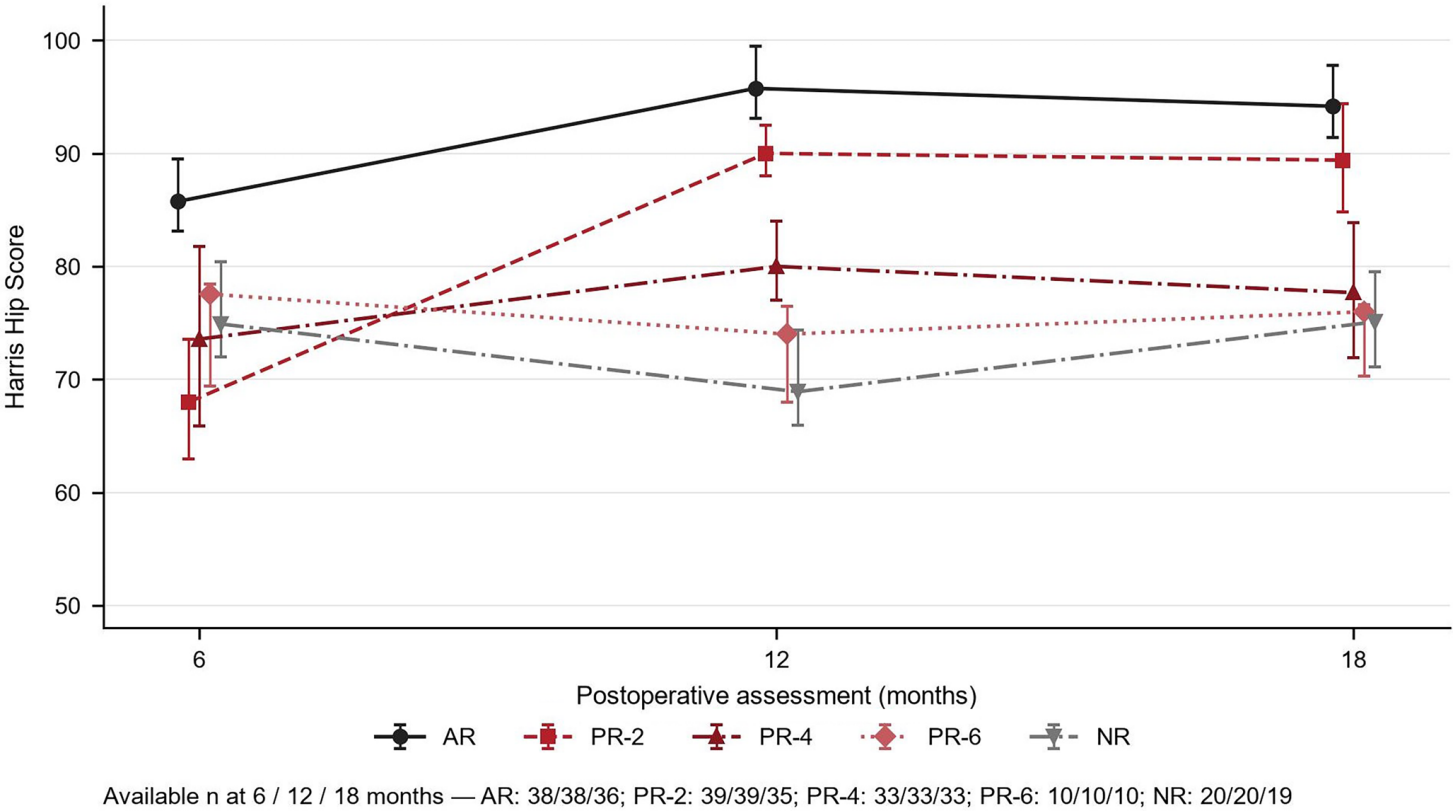
Longitudinal Harris Hip Score (HHS) summaries in the Garden I–III cohort. Markers show the median and error bars the 25th–75th percentiles (interquartile range, IQR) at each recorded assessment. AR: 38, 38, 36; PR‐2: 39, 39, 35; PR‐4: 33, 33, 33; PR‐6: 10, 10, 10; NR: 20, 20, 19 participants at 6, 12, and 18 months, respectively. AR, anatomical reduction; NR, negative‐buttress reduction; PR, positive‐buttress reduction. PR‐2, residual displacement ≤ 2 mm; PR‐4, > 2–4 mm; PR‐6, > 4 mm.

**FIGURE 6:**
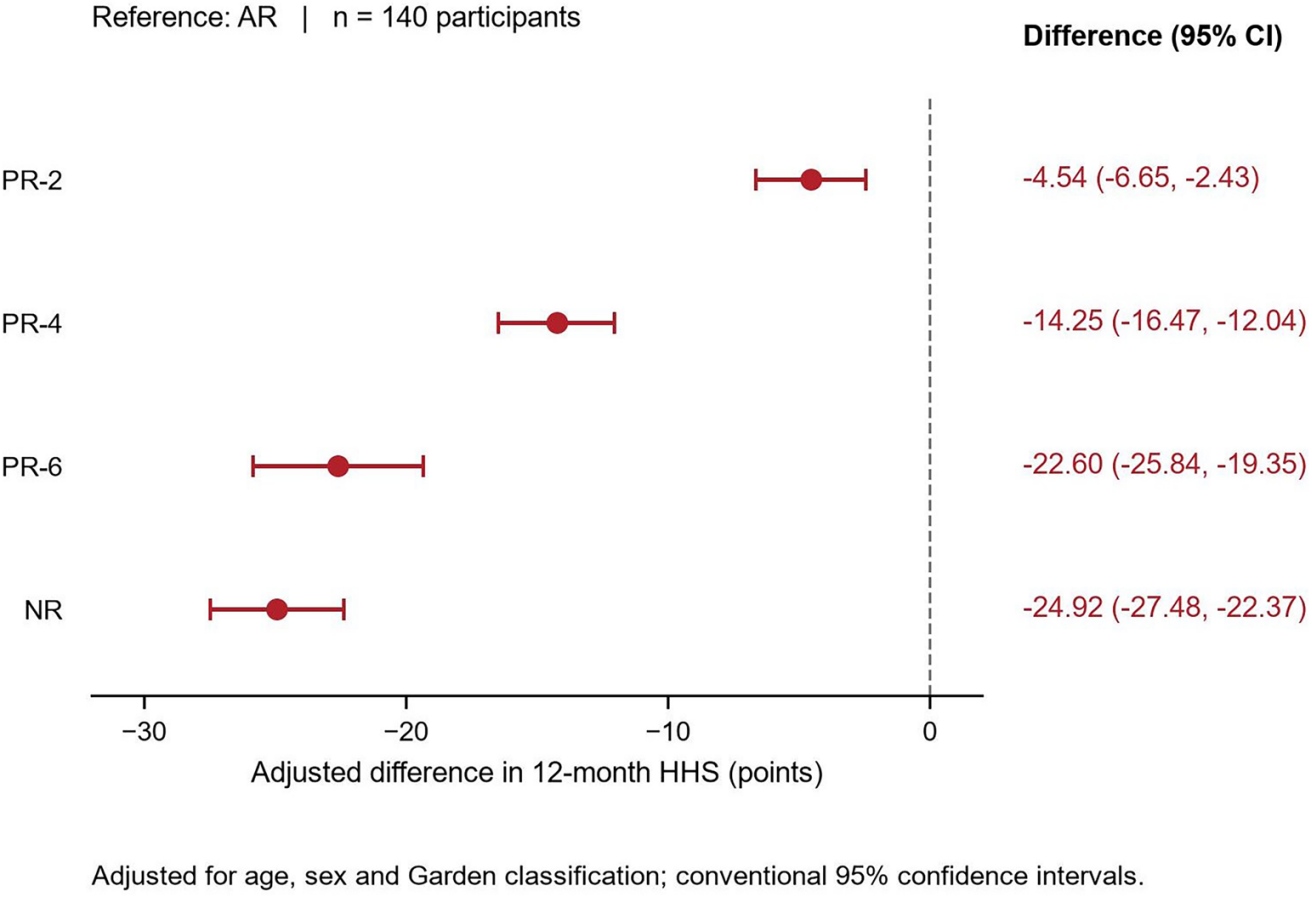
Adjusted between‐group differences in 12‐month Harris Hip Score (HHS) in 140 participants (Garden I–III). Points are ordinary least‐squares regression coefficients relative to anatomical reduction (AR); horizontal bars are conventional two‐sided 95% confidence intervals, using Student's *t* distribution with residual degrees of freedom. Models adjust for age, sex, and categorical Garden classification. The dashed vertical line denotes no difference from AR. NR, negative‐buttress reduction; PR, positive‐buttress reduction; PR‐2, residual displacement ≤ 2 mm; PR‐4, > 2–4 mm; PR‐6, > 4 mm. The plotted estimates correspond to Model 1 in Table 3.

Relative to AR, the difference in 12‐month HHS was −4.54 points (95% CI, −6.65 to −2.43) for PR‐2, −14.25 points (95% CI, −16.47 to −12.04) for PR‐4, −22.60 points (95% CI, −25.84 to −19.35) for PR‐6, and −24.92 points (95% CI, −27.48 to −22.37) for NR (Table 3, Model 1). Expanded adjustment yielded the same ordering, including a PR‐2 versus AR difference of −4.36 points (95% CI, −6.29 to −2.44; Table 3, Model 2). Within the positive buttress subgroup, each 1‐category increase in displacement (PR‐2 to PR‐4 to PR‐6) was associated with a 9.43‐point decrease in 12‐month HHS (95% CI, −11.03 to −7.82; Table 6 and Figure 7).

**TABLE 6:** Exploratory ordinal‐category trend analysis within positive‐buttress reduction only (*n* = 82).

	Adjusted difference in 12‐month HHS, points	95% CI	*p*
Per 1‐category increase in positive‐buttress displacement (PR‐2 → PR‐4 → PR‐6)	−9.43	−11.03 to −7.82	< 0.001

*Note:* Multivariable linear regression adjusted for corrected age, sex, and categorical Garden classification; conventional *t*‐based 95% CIs are shown. The coefficient is the difference per ordinal PR category, not per millimeter and not relative to AR. Continuous‐displacement models are reported separately in Table S1.

Abbreviations: CI, confidence interval; HHS, Harris Hip Score; PR‐2, positive‐buttress displacement ≤ 2 mm; PR‐4, > 2–4 mm; PR‐6, > 4 mm.

**FIGURE 7:**
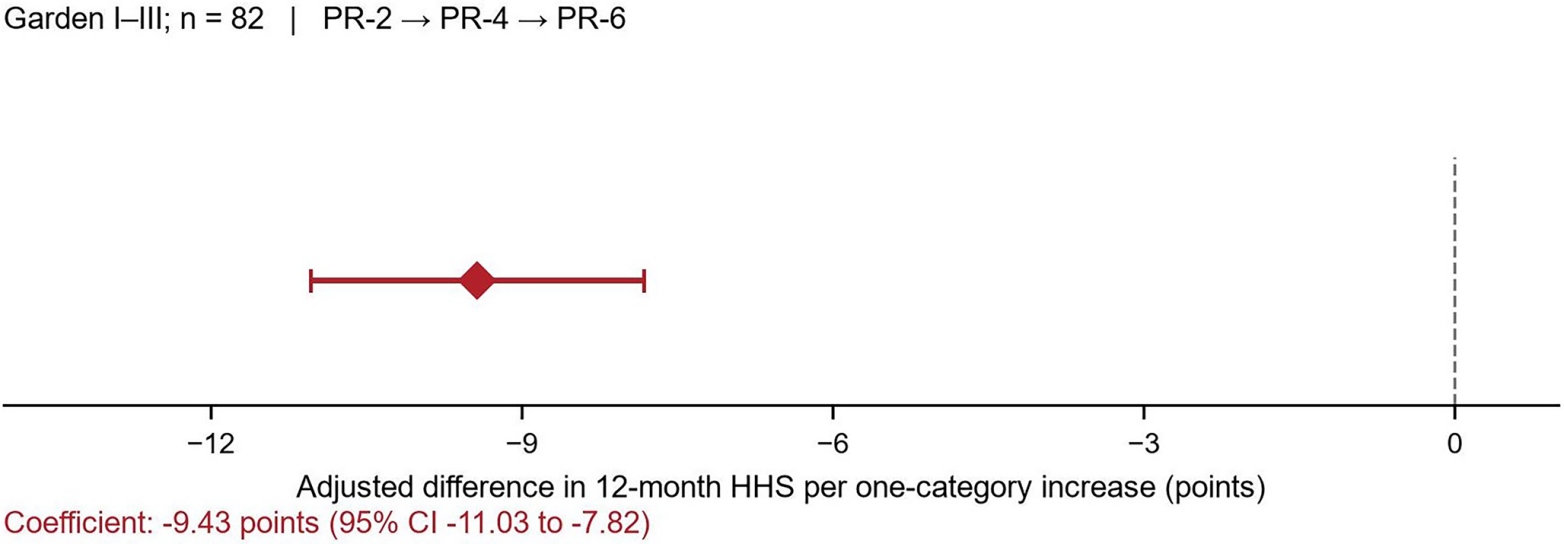
Ordinal association between positive‐buttress displacement categories and 12‐month Harris Hip Score (HHS) after exclusion of Garden IV fractures. The model includes 82 participants with positive‐buttress reduction (PR) and adjusts for age, sex, and categorical Garden classification. The diamond denotes the change in HHS per one‐category increase from PR‐2 (≤ 2 mm) to PR‐4 (> 2–4 mm) to PR‐6 (> 4 mm). The dashed vertical line indicates zero ordinal association.

Secondary surgery again occurred only in the NR group (2 of 20, 10.0%; *p* = 0.024). No secondary surgery or AVN was observed in the PR‐2 subgroup. AVN occurred in one patient each in the PR‐4, PR‐6, and NR groups. Femoral neck shortening was documented in one patient in AR, none in PR‐2, one in PR‐4, one in PR‐6, and two in NR (Table 5).

### 3.6 Sensitivity Analyses

The association direction was retained with HC3 inference, median quantile regression, restriction to Garden II‐III fractures, five‐category ordinal modeling, and generalized overlap weighting. Full alternative estimates and inference definitions are provided in Table S10.

#### 3.6.1 Continuous Displacement and Inclusion of Garden IV Fractures

Continuous modeling yielded lower 12‐month HHS with greater residual positive displacement among all 98 PR cases (−2.93 points per millimeter; HC3 95% CI, −3.78 to −2.07; *p* < 0.001) and among the 82 PR cases with Garden I‐III fractures (−3.89; −4.66 to −3.12; *p* < 0.001). These associations were directionally consistent with the categorical comparisons and did not depend on assigning patients to the selected 2‐ and 4‐mm categories (Table S1 and Figure S3). The five‐group sensitivity analysis retaining all 40 Garden IV fractures also showed negative associations for each nonanatomical group versus AR, with both base and expanded models reported in Table S2 and Figure S4.

#### 3.6.2 Additional Clinical Covariates and Surgeon Analyses

Base and expanded clinical results are presented side by side in Tables 2 and 3. Implant‐position and surgeon analyses, including fixed effects and leave‐one‐surgeon‐out refitting, retained negative point estimates for each nonanatomical group versus AR (Tables S3 and S4). The primary‐surgeon case‐count detail is provided in Table S11.

#### 3.6.3 Sensitivity Analysis Restricted to Patients Younger Than 60 Years

The age‐restricted analysis retained 160 patients (mean age, 38.5 ± 11.7 years; range, 18–59), excluding 20 patients aged 61–70 years. The expanded overall model yielded differences versus AR of −9.96 points for PR (95% CI, −12.12 to −7.80) and −25.43 for NR (−27.52 to −23.35), compared with −9.55 and −25.36 points in the corresponding age‐unrestricted model. After restriction to both age < 60 and Garden I‐III fractures (*n* = 121), the expanded PR‐2 versus AR difference was −5.06 points (−7.35 to −2.78), compared with −4.36 points in all 140 Garden I–III cases. Complete matched‐model comparisons are reported in Table S12.

#### 3.6.4 Open Reduction and Rehabilitation Comparisons

In the Garden I–III five‐group analysis, open reduction occurred in 2/38 AR cases (5.3%), 8/39 PR‐2 cases (20.5%), 3/33 PR‐4 cases (9.1%), 1/10 PR‐6 cases (10.0%), and 4/20 NR cases (20.0%). For PR ≤ 2 mm versus > 2 mm, the conditional odds ratio for open reduction was 2.49 (95% CI, 0.60–12.37; *p* = 0.213) in the restricted PR cohort and 2.06 (0.54–8.70; *p* = 0.248) in all PR cases. Thus, a higher open‐reduction requirement for the smaller‐offset group was not established, but the wide intervals do not exclude an important difference (Tables 5 and S5 and Figure S5). In the restricted five‐group comparison, non‐weightbearing, bilateral‐crutch, and single‐crutch records differed between groups after Holm correction (*p* < 0.001, < 0.001, and 0.003), whereas the full‐weightbearing field did not (*p* = 0.131; Table S6). Rehabilitation‐HHS correlations, one‐field‐at‐a‐time adjusted models, and paired longitudinal summaries are presented in Tables S7–S9.

## 4 Discussion

### 4.1 Principal Findings

At the 12‐month primary endpoint, postoperative function after FNS fixation was associated with reduction pattern. AR had the highest HHS, NR the lowest, and PR an intermediate but heterogeneous profile. In the exploratory analysis restricted to Garden I–III fractures, HHS was lower in larger positive‐buttress displacement categories. The categorical and continuous analyses were consistent in direction. Their agreement supports an association between larger residual displacement and lower 12‐month HHS. It does not identify an abrupt change at 2 or 4 mm or establish either boundary as clinical treatment cutoffs. The additional all‐Garden analysis showed the same direction. Garden IV fractures and open reduction were unevenly distributed across patterns; adjustment cannot eliminate residual confounding from fracture complexity, reduction difficulty, surgeon/team decisions, and unmeasured factors. Thus, the observed association does not show that changing reduction pattern or performing open reduction improves function.

The adjusted PR‐2 versus AR difference was modest at 4.54 points, while the median 12‐month HHS values were 90.0 and 95.8. Both medians fall within the conventionally described excellent range of 90–100 [20, 24]. He et al. reported a mean HHS of 90.42 after FNS fixation, with complications in two of 33 patients [20]. In another FNS cohort, Jiang et al. found no statistically detectable difference in HHS between reduction groups [25]. In our restricted cohort, neither AR nor PR‐2 had secondary surgery or AVN, and femoral neck shortening occurred in one AR patient and no PR‐2 patients. Taken together, the small score difference and low observed complication counts do not establish clinically important functional inferiority of PR‐2. A small positive offset may preserve inferomedial cortical abutment for controlled impaction, whereas larger displacement may reduce cortical contact, shift load transfer eccentrically, and increase sliding, shortening, and functional loss [10, 11, 12, 13, 14, 15, 27, 28, 29, 30]. Beyond a small range, the construct may no longer act as an effective buttress and may approach the disadvantage of NR.

The 6‐month findings do not support uniformly ordered recovery. Recorded rehabilitation differed between groups, and several rehabilitation fields were associated with HHS or subsequent score change. This is consistent with rehabilitation being a potentially relevant source of variation, as postoperative loading has also been studied in younger adults after femoral neck fracture fixation [8]. However, the retrospective associations cannot determine whether rehabilitation caused the nonmonotonic trajectory or was adapted in response to recovery. The small PR‐6 subgroup, individual variation, and differences among treating physicians may contribute. HHS assessor blinding reduced bias related to knowledge of reduction pattern, but does not remove other sources of measurement variation. Without dated complication records, the contribution of complication timing cannot be evaluated.

### 4.2 Treatment Selection and Confounding

Garden classification, open reduction, and surgeon/team factors are clinically entangled with fracture complexity and reduction difficulty. Implant choice does not neutralize reduction morphology. Comparative studies have evaluated FNS against dynamic hip screw and cannulated screw constructs, but those studies primarily address implant selection rather than the residual reduction morphology assessed here [31, 32, 33]. The additional clinical‐covariate, implant‐position, and surgeon sensitivity analyses retained the direction of the 12‐month group differences. These conditional associations are more extensively adjusted than the original models but remain subject to treatment selection, uncertainty in recorded exposure definitions, and unmeasured confounding.

### 4.3 Strengths and Limitations

This study has strengths, including a single FNS construct, blinded HHS and radiographic assessment, three independent radiographic evaluators, a prespecified 12‐month endpoint, and sensitivity analyses. The retrospective design and clinician‐selected FNS use introduce selection bias and preclude causal interpretation. The principal five‐group displacement analysis concerns Garden I‐III fractures; the all‐Garden sensitivity analysis is supportive but does not provide a separately powered or validated Garden IV estimate. Eligibility had no upper age limit, but the cohort was predominantly young or middle‐aged (mean 41.6 ± 14.1 years; range 18–70 years), reflecting clinicians' selection of FNS treatment. The additional analysis restricted to patients younger than 60 years showed the same direction of association. This sensitivity analysis does not remove treatment‐selection bias, establish equivalent effects across ages, or make 60 years a universal boundary for choosing arthroplasty; applicability to older patients remains limited. Anteroposterior and lateral radiographs cannot fully characterize rotation, posterior tilt, or other three‐dimensional deformities; a lateral angulation limit of 15° does not exclude such abnormalities. Future prospective studies should consider CT or validated three‐dimensional assessment methods, where clinically appropriate. Individualized rehabilitation, uncertainty about the timing and definitions of some retrospectively recorded fields, and unavailable patient‐level ward assignments leave residual confounding. Surgeon analyses involved small and uneven clusters and cannot remove all operator‐related effects. Image review was retrospective; reciprocal masking of HHS assessors and image evaluators reduces, but does not eliminate, assessment bias. Complication events were few and their occurrence dates were unavailable. The PR‐6 subgroup was small, and the exploratory category and continuous analyses do not establish a clinically actionable displacement threshold. HHS‐based findings remain provisional pending verification of exact cross‐time and HHS‐rehabilitation relationships against the original assessments (Table S7).

## 5 Conclusions

In conclusion, reduction pattern was associated with 12‐month HHS after FNS fixation. These observational associations do not show that changing reduction pattern or performing open reduction improves function. In the exploratory Garden I–III analysis, PR‐2 had the highest 12‐month HHS among the nonanatomical groups, whereas larger positive‐buttress displacement categories had lower scores. Continuous‐displacement and all‐Garden sensitivity analyses supported the direction of association. The modest PR‐2 versus AR difference does not establish clinically important functional inferiority. PR‐2, PR‐4, and PR‐6 were exploratory categories rather than validated clinical thresholds or indications for additional reduction or open surgery.

## Author Contributions

**Jian Xing:** conceptualization, methodology, formal analysis, investigation, data curation, writing – original draft. **Yanan Ren:** investigation, data curation, writing – review and editing. **Pengfei Wang:** conceptualization, methodology, visualization, software, project administration. **Kun Zhang:** writing – review and editing.

## Funding

This work was supported by the General Medical Project of Xi'an Science and Technology Bureau (25YXYJYB00113).

## Ethics Statement

This retrospective study was approved by the Ethics Committee of Honghui Hospital, Xi'an Jiaotong University (approval number 2026‐KY‐032‐01) and was conducted in accordance with the Declaration of Helsinki and its later amendments.

## Consent

The requirement for informed consent was waived because of the retrospective study design.

## Conflicts of Interest

The authors declare no conflicts of interest.

## Supporting information


**Table S1:** Continuous residual displacement and curvature.
**Table S2:** Five‐group analysis retaining all Garden IV fractures.
**Table S3:** Expanded clinical and implant sensitivity models.
**Table S4:** Individual clinical factors and surgeon sensitivity.
**Table S5:** Open reduction rates and smaller‐offset comparisons.
**Table S6:** Recorded rehabilitation variables by reduction group.
**Table S7:** Rehabilitation records and functional outcomes.
**Table S8:** Postoperative rehabilitation sensitivity coefficients.
**Table S9:** Paired HHS change and longitudinal interaction.
**Table S10:** Recomputed previously described sensitivity analyses.
**Table S11:** Primary surgeon case counts.
**Table S12:** Age restriction below 60 years.
**Figure S1:** Individual Harris Hip Score (HHS) distributions in the Garden I–IV cohort. Panels (a–c) show the recorded 6‐, 12‐, and 18‐month assessments, respectively. Each small symbol denotes one participant at that assessment. Large horizontal bars mark group medians; vertical bars span the 25th–75th percentiles (interquartile range, IQR). Sample sizes appear beneath group labels. Missing 18‐month records are omitted from that assessment only. AR, anatomical reduction; NR, negative‐buttress reduction; PR, positive‐buttress reduction. The figure is descriptive and does not encode significance tests.
**Figure S2:** Individual Harris Hip Score (HHS) distributions in the Garden I–III cohort. Panels (a–c) show the recorded 6‐, 12‐, and 18‐month assessments, respectively. Each small symbol denotes one participant at that assessment. Large horizontal bars mark group medians; vertical bars span the 25th–75th percentiles (interquartile range, IQR). Sample sizes appear beneath group labels. Missing 18‐month records are omitted from that assessment only. AR, anatomical reduction; NR, negative‐buttress reduction; PR, positive‐buttress reduction. PR‐2, ≤ 2 mm; PR‐4, > 2–4 mm; PR‐6, > 4 mm residual displacement. The figure is descriptive and does not encode significance tests.
**Figure S3:** Continuous residual positive displacement and 12‐month Harris Hip Score (HHS). Panel (a) includes all 98 participants with positive‐buttress reduction (PR), including Garden IV fractures and panel (b) includes the 82 PR participants with Garden I–III fractures. Gray points are individual observed HHS values without covariate adjustment. Red lines are linear‐model estimated marginal means standardized to the age, sex and categorical Garden distribution of each respective PR cohort. Shaded bands are two‐sided 95% confidence intervals for these standardized means, using HC3 covariance and Student's *t* distribution with residual degrees of freedom. The models estimate an association per millimeter and do not identify or validate a clinical threshold. All eligible PR observations are retained.
**Figure S4:** Five‐group 12‐month Harris Hip Score (HHS) sensitivity analysis retaining Garden IV fractures (*n* = 180). Points show adjusted differences relative to anatomical reduction (AR); horizontal bars are two‐sided 95% confidence intervals. Black circles show the base model adjusting for age, sex and categorical Garden classification. Gray squares additionally adjust for open reduction, injury‐to‐surgery interval, Pauwels angle, posterior tilt, body mass index and smoking category. These two models use HC3 covariance and Student's t distribution with residual degrees of freedom. Red diamonds show the same expanded point estimates with surgeon‐clustered CR1 covariance, small‐sample correction and *t*‐based inference over 22 degrees of freedom (23 surgeon codes). Clustering addresses correlated residuals, not surgeon‐level confounding by itself. NR, negative‐buttress reduction; PR‐2, residual positive displacement ≤ 2 mm; PR‐4, > 2–4 mm; PR‐6, > 4 mm.
**Figure S5:** Open‐reduction proportions across the five reduction groups. Panel (a) includes all 180 participants (Garden I–IV); panel (b) includes 140 participants after exclusion of Garden IV fractures. Bar heights are observed percentages, and labels show the number undergoing open reduction divided by the group denominator. Global two‐sided Fisher–Freeman–Halton exact tests compare the five groups separately within each cohort. AR, anatomical reduction; NR, negative‐buttress reduction; PR‐2, positive‐buttress displacement ≤ 2 mm; PR‐4, > 2–4 mm; PR‐6, > 4 mm.

## Data Availability

The data that support the findings of this study are available from the corresponding author upon reasonable request.

## References

[1] G. P. Slobogean , D. J. Stockton , B. Zeng , D. Wang , B. T. Ma , and A. N. Pollak , “Femoral Neck Fractures in Adults Treated With Internal Fixation: A Prospective Multicenter Chinese Cohort,” Journal of the American Academy of Orthopaedic Surgeons 25, no. 4 (2017): 297–303. 10.5435/JAAOS-D-15-00661 28248692

[2] FAITH Investigators , “Fracture Fixation in the Operative Management of Hip Fractures (FAITH): An International, Multicentre, Randomised Controlled Trial,” Lancet 389, no. 10078 (2017): 1519–1527. 10.1016/S0140-6736(17)30066-1 PMC5597430 28262269

[3] S. Sprague , E. H. Schemitsch , M. Swiontkowski , et al., “Factors Associated With Revision Surgery After Internal Fixation of Hip Fractures,” Journal of Orthopaedic Trauma 32, no. 5 (2018): 223–230. 10.1097/BOT.0000000000001162 PMC5986292 29677091

[4] J. Felton , G. P. Slobogean , S. S. Jackson , et al., “Femoral Neck Shortening After Hip Fracture Fixation Is Associated With Inferior Hip Function: Results From the FAITH Trial,” Journal of Orthopaedic Trauma 33, no. 10 (2019): 487–496. 10.1097/BOT.0000000000001551 31464855

[5] J. T. Patterson , K. Ishii , P. Tornetta, 3rd , et al., “Open Reduction Is Associated With Greater Hazard of Early Reoperation After Internal Fixation of Displaced Femoral Neck Fractures in Adults 18 to 65 Years,” Journal of Orthopaedic Trauma 34, no. 6 (2020): 294–301. 10.1097/BOT.0000000000001711 32079891

[6] S. H. Wang , J. J. Yang , H. C. Shen , L. C. Lin , M. S. Lee , and R. Y. Pan , “Using a Modified Pauwels Method to Predict the Outcome of Femoral Neck Fracture in Relatively Young Adults,” Injury 46, no. 10 (2015): 1969–1974. 10.1016/j.injury.2015.06.016 26113033

[7] Z. Hua , X. Zhang , X. Li , et al., “Association Between Surgical Delays for Femoral Neck Fractures and Early Postoperative Complications in Young and Middle‐Aged Adults: A Study Based on the National Inpatient Sample Database,” Injury 55, no. 10 (2024): 111690. 10.1016/j.injury.2024.111690 39002323

[8] K. Hasegawa , Y. Takegami , K. Tokutake , et al., “Early Versus Delayed Weight Bearing After Internal Fixation for Femoral Neck Fracture in Younger Adults: A Multicenter Retrospective Study,” Injury 55, no. 2 (2024): 111292. 10.1016/j.injury.2023.111292 38157758

[9] P. Sjöholm , V. Otten , O. Wolf , et al., “Posterior and Anterior Tilt Increases the Risk of Failure After Internal Fixation of Garden I and II Femoral Neck Fracture,” Acta Orthopaedica 90, no. 6 (2019): 537–541. 10.1080/17453674.2019.1637469 PMC6844400 31269853

[10] Y. Gotfried , S. Kovalenko , and D. Fuchs , “Nonanatomical Reduction of Displaced Subcapital Femoral Fractures (Gotfried Reduction),” Journal of Orthopaedic Trauma 27, no. 11 (2013): e254–e259. 10.1097/BOT.0b013e31828f8ffc 23481921

[11] W. F. Xiong , S. M. Chang , Y. Q. Zhang , S. J. Hu , and S. C. Du , “Inferior Calcar Buttress Reduction Pattern for Displaced Femoral Neck Fractures in Young Adults: A Preliminary Report and an Effective Alternative,” Journal of Orthopaedic Surgery and Research 14, no. 1 (2019): 70. 10.1186/s13018-019-1109-x PMC6396447 30819226

[12] K. Huang , X. Fang , G. Li , and J. Yue , “Assessing the Effect of Gotfried Reduction With Positive Buttress Pattern in the Young Femoral Neck Fracture,” Journal of Orthopaedic Surgery and Research 15, no. 1 (2020): 511. 10.1186/s13018-020-02039-0 PMC7648971 33160395

[13] Y. Wang , J. X. Ma , T. Yin , Z. Han , S. S. Cui , and Z. P. Liu , “Correlation Between Reduction Quality of Femoral Neck Fracture and Femoral Head Necrosis Based on Biomechanics,” Orthopaedic Surgery 11, no. 2 (2019): 318–324. 10.1111/os.12458 PMC6594541 31025811

[14] G. Wang , B. Wang , Y. Tang , and H. L. Yang , “A Quantitative Biomechanical Study of Positive Buttress Techniques for Femoral Neck Fractures: A Finite Element Analysis,” Chinese Medical Journal 132, no. 21 (2019): 2588–2593. 10.1097/CM9.0000000000000490 PMC6846255 31658158

[15] G. Zhao , M. Liu , B. Li , H. Sun , and B. Wei , “Clinical Observation and Finite Element Analysis of Cannulated Screw Internal Fixation in the Treatment of Femoral Neck Fracture Based on Different Reduction Quality,” Journal of Orthopaedic Surgery and Research 16, no. 1 (2021): 450. 10.1186/s13018-021-02580-6 PMC8276405 34256786

[16] K. Stoffel , I. Zderic , F. Gras , et al., “Biomechanical Evaluation of the Femoral Neck System in Unstable Pauwels III Femoral Neck Fractures: A Comparison With the Dynamic Hip Screw and Cannulated Screws,” Journal of Orthopaedic Trauma 31, no. 3 (2017): 131–137. 10.1097/BOT.0000000000000739 27755333

[17] C. Schopper , I. Zderic , J. Menze , et al., “Higher Stability and More Predictive Fixation With the Femoral Neck System Versus Hansson Pins in Femoral Neck Fractures Pauwels II,” Journal of Orthopaedic Translation 24 (2020): 88–95. 10.1016/j.jot.2020.06.002 PMC7387742 32775200

[18] O. Vazquez , A. Gamulin , D. Hannouche , and W. Belaieff , “Osteosynthesis of Non‐Displaced Femoral Neck Fractures in the Elderly Population Using the Femoral Neck System: Short‐Term Clinical and Radiological Outcomes,” Journal of Orthopaedic Surgery and Research 16, no. 1 (2021): 477. 10.1186/s13018-021-02622-z PMC8336369 34348753

[19] Y. Tang , Z. Zhang , L. Wang , W. Xiong , Q. Fang , and G. Wang , “Femoral Neck System Versus Inverted Cannulated Cancellous Screw for the Treatment of Femoral Neck Fractures in Adults: A Preliminary Comparative Study,” Journal of Orthopaedic Surgery and Research 16, no. 1 (2021): 504. 10.1186/s13018-021-02659-0 PMC8365927 34399801

[20] C. He , Y. Lu , Q. Wang , et al., “Comparison of the Clinical Efficacy of a Femoral Neck System Versus Cannulated Screws in the Treatment of Femoral Neck Fracture in Young Adults,” BMC Musculoskeletal Disorders 22, no. 1 (2021): 994. 10.1186/s12891-021-04888-0 PMC8630835 34844578

[21] X. Q. Zhou , Z. Q. Li , R. J. Xu , et al., “Comparison of Early Clinical Results for Femoral Neck System and Cannulated Screws in the Treatment of Unstable Femoral Neck Fractures,” Orthopaedic Surgery 13, no. 6 (2021): 1802–1809. 10.1111/os.13098 PMC8523763 34351048

[22] Y. Nibe , T. Matsumura , T. Takahashi , T. Kubo , Y. Matsumoto , and K. Takeshita , “A Comparison Between the Femoral Neck System and Other Implants for Elderly Patients With Femoral Neck Fracture: A Preliminary Report of a Newly Developed Implant,” Journal of Orthopaedic Science 27, no. 4 (2022): 876–880. 10.1016/j.jos.2021.04.016 34090779

[23] R. C. Stassen , R. M. Jeuken , B. Boonen , B. Meesters , E. R. de Loos , and R. van Vugt , “First Clinical Results of 1‐Year Follow‐Up of the Femoral Neck System for Internal Fixation of Femoral Neck Fractures,” Archives of Orthopaedic and Trauma Surgery 142, no. 12 (2022): 3755–3763. 10.1007/s00402-021-04216-0 34734328

[24] K. Stoffel , C. Michelitsch , R. Arora , et al., “Clinical Performance of the Femoral Neck System Within One Year in 125 Patients With Acute Femoral Neck Fractures: A Prospective Observational Case Series,” Archives of Orthopaedic and Trauma Surgery 143, no. 7 (2023): 4155–4164. 10.1007/s00402-022-04686-w PMC10293436 36460761

[25] Q. Jiang , Y. Liu , X. Bai , et al., “Nonanatomical Reduction of Femoral Neck Fractures in Young Patients Treated With Femoral Neck System: A Retrospective Cohort Study,” BMC Musculoskeletal Disorders 24, no. 1 (2023): 412. 10.1186/s12891-023-06551-2 PMC10207767 37226140

[26] J. Y. Yoon , S. E. Byun , and Y. H. Cho , “Fixation of Femoral Neck Fracture With Femoral Neck System: A Retrospective Cohort Study of 43 Patients,” BMC Musculoskeletal Disorders 25, no. 1 (2024): 8. 10.1186/s12891-023-07113-2 PMC10759579 38166882

[27] L. Chen , J. B. Jiang , H. Ma , X. Duan , and J. L. Chen , “Factors Associated With Early Failure of the Femoral Neck System in Patients With Femoral Neck Fractures,” BMC Musculoskeletal Disorders 24, no. 1 (2023): 912. 10.1186/s12891-023-06994-7 PMC10680198 38012667

[28] S. Weng , D. Lin , J. Zeng , et al., “Optimal Sliding Distance in Femoral Neck System for Displaced Femoral Neck Fractures: A Retrospective Cohort Study,” Journal of Orthopaedic Surgery and Research 19, no. 1 (2024): 690. 10.1186/s13018-024-05190-0 PMC11515223 39456012

[29] D. Lin , W. Gong , C. Lin , et al., “Anti‐Shortening Screw for the Prevention of Postoperative Shortening in Displaced Femoral Neck Fractures: A Retrospective Cohort Study,” Orthopaedic Surgery 16, no. 12 (2024): 2967–2975. 10.1111/os.14232 PMC11608781 39219035

[30] D. Lin , Y. Liang , P. Chen , S. Zheng , and F. Lin , “Pre‐Sliding Technique to Improve Femoral Neck System Against Shortening: A Retrospective Cohort Study,” BMC Musculoskeletal Disorders 25, no. 1 (2024): 293. 10.1186/s12891-024-07391-4 PMC11020420 38627701

[31] K. Schuetze , J. Burkhardt , C. Pankratz , et al., “Is New Always Better: Comparison of the Femoral Neck System and the Dynamic Hip Screw in the Treatment of Femoral Neck Fractures,” Archives of Orthopaedic and Trauma Surgery 143, no. 6 (2023): 3155–3161. 10.1007/s00402-022-04551-w PMC10191950 35867115

[32] S. G. Yan , Y. Cui , D. Li , F. Liu , X. Hua , and F. Schmidutz , “Femoral Neck System Versus Three Cannulated Screws for Fixation of Femoral Neck Fractures in Younger Patients: A Retrospective Cohort Study,” Journal of Investigative Surgery 36, no. 1 (2023): 2266752. 10.1080/08941939.2023.2266752 37813399

[33] Z. Xu , J. Sun , J. Li , et al., “Comparative Analysis of the Femoral Neck System Versus Cannulated Cancellous Screws in the Treatment of Middle‐Aged and Elderly Patients With Femoral Neck Fractures: Clinical Outcomes and Biomechanical Insights,” BMC Musculoskeletal Disorders 25, no. 1 (2024): 735. 10.1186/s12891-024-07863-7 PMC11401286 39277709

