# Supplementary material for: Residual Positive Buttress Displacement After Femoral Neck System Fixation for Acute Femoral Neck Fractures: A Retrospective Cohort Study
Source: Orthop Surg. 2026 Oct 22:10.1111/os.70450. Online ahead of print. doi: 10.1111/os.70450 (PMC13691225; doi:10.1111/os.70450)
Supplement: Supplementary file 1 [file OS-9999-0-s001.docx]

Supplementary Statistical Analyses

Manuscript 9690723 ·

# Supplementary Methods

Base models adjusted for age, sex, and categorical Garden classification. Expanded models additionally adjusted for open versus closed reduction, injury-to-surgery interval in days, recorded Pauwels angle and posterior tilt in degrees, BMI, and smoking (never, former, current). Sensitivity models additionally included recorded tip-apex distance and central versus eccentric implant position. All antirotation screws were standard FNS components, and no additional screw was used.

Model hierarchy and reporting locations. The overall three-group analysis is primary; the Garden I-III five-group analysis is exploratory. Each is reported with Model 1 (base) and Model 2 (expanded) in main Tables 4 and 5. Supplementary Table S2 retains the all-Garden five-group sensitivity analysis, S1 the continuous PR analysis, S3-S10 the other sensitivity analyses, S11 the primary-surgeon counts, and S12 the age-restricted comparisons. Excluding Garden IV and excluding age ≥60 change the analysis population; base versus expanded changes the covariate set.

Linear-regression HC3 confidence intervals and two-sided tests used the t distribution with n-p residual degrees of freedom. Surgeon-clustered inference used the finite-sample CR1 correction, with t(G-1); there were 23 surgeon codes in the group models. Clustering changes uncertainty estimates but does not itself control surgeon-level confounding. Therefore, expanded models were also refitted with surgeon fixed effects, and with each surgeon omitted in turn. Ward membership was unavailable and was not inferred from surgeon codes.

Continuous PR models used the mean of the three recorded displacement measurements, in millimeters, among PR cases only. Linear slopes were estimated in all 98 PR cases and the 82 Garden I-III PR cases. An additional centered squared-displacement term examined curvature; no breakpoint optimization or data-selected cutoff was performed. Figure S3 presents adjusted marginal means standardized to the covariate distribution of each PR population, with 95% confidence intervals for the mean.

The continuous slope describes the conditional HHS association per millimeter within PR; category contrasts describe differences between reduction groups. Directional agreement is complementary evidence from the same records and does not identify or validate a 2- or 4-mm clinical breakpoint.

Open-reduction rates across three or five groups were tested using exact two-sided Fisher-Freeman-Halton enumeration with fixed margins. PR ≤2 mm versus >2 mm was assessed by Fisher’s exact test, reporting the conditional maximum-likelihood odds ratio and an exact conditional 95% interval. Each comparison is reported.

The four rehabilitation records are summarized in weeks as mean ± SD. Bilateral-crutch use is a duration. Group comparisons used tie-corrected Kruskal-Wallis tests, with Holm adjustment across four fields within each population. Spearman associations with 6-month HHS, 12-month HHS, and individual 6-to-12-month HHS change used Holm adjustment within each population/stratum set of 12 comparisons; undefined correlations for constant outcomes were omitted from adjustment and reported as not estimable. Each rehabilitation field was added separately to the expanded model, both for 12-month HHS and for paired change. These are postoperative sensitivity analyses, not estimates of a rehabilitation treatment effect.

Unadjusted HHS group comparisons at 6, 12, and 18 months used tie-corrected Kruskal-Wallis tests with available cases at each visit; these exploratory P values were not corrected for multiplicity. Longitudinal models included categorical visit, reduction group, their interaction, age, sex, and Garden classification, with patient-clustered CR1 standard errors. Joint group-by-time interactions used an F test. Available observations were used for the 18-month analysis.

Additional previously described sensitivity analyses were recomputed after the age corrections. Median quantile regression used quantile 0.5, robust covariance, an Epanechnikov kernel, and Hall-Sheather bandwidth. Generalized overlap weights were estimated by multinomial logistic regression on age, sex, and Garden class, using h=1/Σ(1/e_k) and w=h/e_observed. Weighted HC3 intervals treated the estimated weights as fixed and therefore do not include uncertainty from propensity-score estimation. All regression results and secondary open-reduction comparisons are exploratory.

An additional sensitivity analysis requested during revision was restricted to patients younger than 60 years. The original eligibility criteria and 180-patient cohort were unchanged. Base and expanded models were refitted within the same three-group, five-group, and PR-only populations, retaining continuous age as a covariate. Age-unrestricted and age-restricted estimates were compared using identical covariate sets and HC3 t-based inference (Supplementary Table S12). Because the restricted cohort is nested within the full cohort, the comparison was descriptive; no independent-sample test or equivalence claim was made. All 20 new or matched reference fits were full rank and retained every eligible patient without additional missing-data exclusions. Full formulas, coefficients, case counts, leverage, and independent QR/HC3 verification are included in the Under60 reproducibility files.

Software: Python 3.12.14, NumPy 2.3.5, pandas 3.0.1, SciPy 1.18.1, statsmodels 0.15.0, Patsy 1.0.3, and openpyxl 3.1.5.

## Supplementary Table S1 Continuous residual displacement and curvature

| **Population and model** | **n** | **Coefficient** | **HHS difference (95% CI)** | **P** |
| --- | --- | --- | --- | --- |
| All PR · Base linear | 98 | PR displacement per mm | -2.93 (-3.78 to -2.07) | <0.001 |
| All PR · Expanded linear | 98 | PR displacement per mm | -2.82 (-3.75 to -1.88) | <0.001 |
| All PR · Implant-adjusted linear | 98 | PR displacement per mm | -2.62 (-3.53 to -1.72) | <0.001 |
| All PR · Expanded surgeon-clustered | 98 | PR displacement per mm | -2.82 (-3.92 to -1.72) | <0.001 |
| All PR · Quadratic curvature | 98 | Squared centered displacement | 0.37 (-0.06 to 0.81) | 0.090 |
| Garden I-III PR · Base linear | 82 | PR displacement per mm | -3.89 (-4.66 to -3.12) | <0.001 |
| Garden I-III PR · Expanded linear | 82 | PR displacement per mm | -3.89 (-4.66 to -3.11) | <0.001 |
| Garden I-III PR · Implant-adjusted linear | 82 | PR displacement per mm | -3.74 (-4.59 to -2.89) | <0.001 |
| Garden I-III PR · Expanded surgeon-clustered | 82 | PR displacement per mm | -3.89 (-4.70 to -3.07) | <0.001 |
| Garden I-III PR · Quadratic curvature | 82 | Squared centered displacement | 0.34 (-0.13 to 0.81) | 0.153 |

All linear coefficients are HHS points per additional millimeter. The quadratic row is the centered squared-term coefficient, not a linear slope. Linear and quadratic models adjust for age, sex, and Garden class; expanded and implant models use the additional covariates defined above. HC3 t-based inference except where surgeon clustering is specified.

## Supplementary Table S2 Five-group analysis retaining all Garden IV fractures

| **Model** | **n** | **Comparison** | **HHS difference (95% CI)** | **P** |
| --- | --- | --- | --- | --- |
| Base HC3 | 180 | NR vs AR | -25.87 (-27.79 to -23.95) | <0.001 |
| Base HC3 | 180 | PR-2 vs AR | -4.49 (-6.34 to -2.64) | <0.001 |
| Base HC3 | 180 | PR-4 vs AR | -12.16 (-14.47 to -9.84) | <0.001 |
| Base HC3 | 180 | PR-6 vs AR | -16.83 (-21.96 to -11.70) | <0.001 |
| Expanded HC3 | 180 | NR vs AR | -25.46 (-27.48 to -23.44) | <0.001 |
| Expanded HC3 | 180 | PR-2 vs AR | -4.62 (-6.47 to -2.78) | <0.001 |
| Expanded HC3 | 180 | PR-4 vs AR | -12.36 (-14.76 to -9.96) | <0.001 |
| Expanded HC3 | 180 | PR-6 vs AR | -16.69 (-21.86 to -11.52) | <0.001 |
| Implant HC3 | 180 | NR vs AR | -25.35 (-27.47 to -23.23) | <0.001 |
| Implant HC3 | 180 | PR-2 vs AR | -4.58 (-6.49 to -2.67) | <0.001 |
| Implant HC3 | 180 | PR-4 vs AR | -12.13 (-14.53 to -9.72) | <0.001 |
| Implant HC3 | 180 | PR-6 vs AR | -16.08 (-21.11 to -11.06) | <0.001 |
| Expanded surgeon-clustered | 180 | NR vs AR | -25.46 (-27.30 to -23.62) | <0.001 |
| Expanded surgeon-clustered | 180 | PR-2 vs AR | -4.62 (-6.14 to -3.11) | <0.001 |
| Expanded surgeon-clustered | 180 | PR-4 vs AR | -12.36 (-14.93 to -9.78) | <0.001 |
| Expanded surgeon-clustered | 180 | PR-6 vs AR | -16.69 (-22.85 to -10.53) | <0.001 |

All 180 patients are retained: AR 45, PR-2 45, PR-4 38, PR-6 15, NR 37; 40 have Garden IV fractures. AR is the reference. This is an all-Garden sensitivity analysis, not a separate Garden IV subgroup validation.

## Supplementary Table S3 Expanded clinical and implant sensitivity models

| **Population and model** | **n** | **Comparison** | **HHS difference (95% CI)** | **P** |
| --- | --- | --- | --- | --- |
| Overall 3 groups expanded | 180 | NR vs AR | -25.36 (-27.32 to -23.40) | <0.001 |
| Overall 3 groups expanded | 180 | PR vs AR | -9.55 (-11.54 to -7.56) | <0.001 |
| Overall 3 groups implant | 180 | NR vs AR | -25.22 (-27.39 to -23.05) | <0.001 |
| Overall 3 groups implant | 180 | PR vs AR | -9.26 (-11.24 to -7.27) | <0.001 |
| Overall 3 groups clustered | 180 | NR vs AR | -25.36 (-27.28 to -23.43) | <0.001 |
| Overall 3 groups clustered | 180 | PR vs AR | -9.55 (-11.94 to -7.16) | <0.001 |
| Garden I-III 5 groups expanded | 140 | NR vs AR | -24.73 (-27.17 to -22.29) | <0.001 |
| Garden I-III 5 groups expanded | 140 | PR-2 vs AR | -4.36 (-6.29 to -2.44) | <0.001 |
| Garden I-III 5 groups expanded | 140 | PR-4 vs AR | -14.18 (-16.60 to -11.77) | <0.001 |
| Garden I-III 5 groups expanded | 140 | PR-6 vs AR | -22.29 (-26.73 to -17.85) | <0.001 |
| Garden I-III 5 groups implant | 140 | NR vs AR | -24.70 (-27.18 to -22.22) | <0.001 |
| Garden I-III 5 groups implant | 140 | PR-2 vs AR | -4.37 (-6.34 to -2.40) | <0.001 |
| Garden I-III 5 groups implant | 140 | PR-4 vs AR | -13.98 (-16.39 to -11.56) | <0.001 |
| Garden I-III 5 groups implant | 140 | PR-6 vs AR | -21.80 (-26.27 to -17.32) | <0.001 |
| Garden I-III 5 groups clustered | 140 | NR vs AR | -24.73 (-26.66 to -22.80) | <0.001 |
| Garden I-III 5 groups clustered | 140 | PR-2 vs AR | -4.36 (-5.96 to -2.76) | <0.001 |
| Garden I-III 5 groups clustered | 140 | PR-4 vs AR | -14.18 (-16.14 to -12.23) | <0.001 |
| Garden I-III 5 groups clustered | 140 | PR-6 vs AR | -22.29 (-26.92 to -17.65) | <0.001 |

AR is the reference. Expanded: age, sex, Garden, reduction approach, injury-to-surgery interval, Pauwels angle, posterior tilt, BMI, smoking, tip-apex distance, implant position, surgeon-clustered CR1 uncertainty.

## Supplementary Table S4 Individual clinical factors and surgeon sensitivity

| **Recorded factor** | **Overall 3 groups** | **Garden I-III 5 groups** | **All-Garden 5 groups** |
| --- | --- | --- | --- |
| Open vs closed reduction | -1.065 (-3.903 to 1.772); P 0.460 | -1.713 (-4.330 to 0.905); P 0.198 | -1.848 (-4.250 to 0.554); P 0.131 |
| Injury-to-surgery interval per day | -0.060 (-0.392 to 0.271); P 0.719 | 0.047 (-0.221 to 0.316); P 0.728 | -0.027 (-0.313 to 0.259); P 0.852 |
| Pauwels angle per degree | 0.096 (-0.008 to 0.200); P 0.071 | 0.026 (-0.064 to 0.116); P 0.569 | 0.059 (-0.042 to 0.160); P 0.248 |
| Posterior tilt per degree | 0.079 (-0.069 to 0.227); P 0.295 | -0.052 (-0.187 to 0.083); P 0.449 | 0.044 (-0.088 to 0.177); P 0.510 |
| BMI per kg/m² | 0.303 (0.003 to 0.603); P 0.048 | 0.171 (-0.120 to 0.461); P 0.248 | 0.240 (-0.012 to 0.492); P 0.062 |
| Former vs never smoking | 0.407 (-3.922 to 4.736); P 0.853 | 0.634 (-3.942 to 5.210); P 0.784 | -1.171 (-4.830 to 2.488); P 0.528 |
| Current vs never smoking | -2.094 (-4.968 to 0.780); P 0.152 | -1.268 (-3.771 to 1.235); P 0.318 | -1.747 (-4.083 to 0.590); P 0.142 |
| Tip-apex distance per mm | -0.388 (-0.677 to -0.100); P 0.009 | -0.133 (-0.377 to 0.112); P 0.286 | -0.264 (-0.515 to -0.014); P 0.039 |
| Eccentric vs central implant | 1.600 (-2.052 to 5.252); P 0.388 | 0.332 (-3.239 to 3.904); P 0.854 | 1.022 (-2.224 to 4.268); P 0.535 |

Entries are HHS point estimates and 95% CIs from the implant-adjusted models.

### Surgeon fixed effects

| **Population** | **Comparison** | **HHS difference (95% CI)** | **P** |
| --- | --- | --- | --- |
| Overall three groups | NR vs AR | -24.90 (-27.55 to -22.25) | <0.001 |
| Overall three groups | PR vs AR | -9.89 (-12.39 to -7.40) | <0.001 |
| Garden I-III five groups | NR vs AR | -24.16 (-27.58 to -20.73) | <0.001 |
| Garden I-III five groups | PR-2 vs AR | -4.29 (-6.78 to -1.81) | <0.001 |
| Garden I-III five groups | PR-4 vs AR | -14.27 (-17.13 to -11.42) | <0.001 |
| Garden I-III five groups | PR-6 vs AR | -22.97 (-28.68 to -17.27) | <0.001 |
| All-Garden five groups | NR vs AR | -24.72 (-27.25 to -22.18) | <0.001 |
| All-Garden five groups | PR-2 vs AR | -4.75 (-7.17 to -2.34) | <0.001 |
| All-Garden five groups | PR-4 vs AR | -12.21 (-15.04 to -9.38) | <0.001 |
| All-Garden five groups | PR-6 vs AR | -16.46 (-22.03 to -10.89) | <0.001 |

Surgeon fixed effects add indicator terms for the recorded surgeon codes to the expanded model. All three designs were full rank; HC3 t-based inference was used. This addresses recorded surgeon-level differences conditionally.

### Leave one surgeon out

| **Population** | **Comparison** | **Range of point estimates** | **Fits** | **CIs including zero** |
| --- | --- | --- | --- | --- |
| All-Garden five groups | NR vs AR | -25.95 to -25.11 | 23 | 0 |
| All-Garden five groups | PR-2 vs AR | -4.95 to -4.23 | 23 | 0 |
| All-Garden five groups | PR-4 vs AR | -13.12 to -11.92 | 23 | 0 |
| All-Garden five groups | PR-6 vs AR | -18.57 to -15.18 | 23 | 0 |
| Overall three groups | NR vs AR | -25.86 to -25.00 | 23 | 0 |
| Overall three groups | PR vs AR | -10.02 to -8.82 | 23 | 0 |
| Garden I-III five groups | NR vs AR | -25.36 to -24.33 | 23 | 0 |
| Garden I-III five groups | PR-2 vs AR | -4.78 to -3.95 | 23 | 0 |
| Garden I-III five groups | PR-4 vs AR | -14.80 to -13.75 | 23 | 0 |
| Garden I-III five groups | PR-6 vs AR | -23.44 to -21.00 | 23 | 0 |

The reported range is the range of point estimates across leave-one-surgeon-out fits. Each fit used the expanded model and HC3 t-based uncertainty.

## Supplementary Table S5 Open reduction rates and smaller-offset comparisons

| **Population** | **Reduction group** | **Open reduction n/N (%)** | **Global exact P** |
| --- | --- | --- | --- |
| Overall three groups | AR | 4/45 (8.9%) | 0.012 |
| Overall three groups | PR | 13/98 (13.3%) | 0.012 |
| Overall three groups | NR | 12/37 (32.4%) | 0.012 |
| Garden I-III five groups | AR | 2/38 (5.3%) | 0.227 |
| Garden I-III five groups | PR-2 | 8/39 (20.5%) | 0.227 |
| Garden I-III five groups | PR-4 | 3/33 (9.1%) | 0.227 |
| Garden I-III five groups | PR-6 | 1/10 (10.0%) | 0.227 |
| Garden I-III five groups | NR | 4/20 (20.0%) | 0.227 |
| All-Garden five groups | AR | 4/45 (8.9%) | 0.035 |
| All-Garden five groups | PR-2 | 8/45 (17.8%) | 0.035 |
| All-Garden five groups | PR-4 | 3/38 (7.9%) | 0.035 |
| All-Garden five groups | PR-6 | 2/15 (13.3%) | 0.035 |
| All-Garden five groups | NR | 12/37 (32.4%) | 0.035 |

The global P value is repeated for clarity and is a single comparison per population.

| **Population** | **PR ≤2 mm open/N** | **PR >2 mm open/N** | **Conditional OR (95% CI)** | **P** |
| --- | --- | --- | --- | --- |
| All PR | 8/45 | 5/53 | 2.06 (0.54 to 8.70) | 0.248 |
| Garden I-III PR | 8/39 | 4/43 | 2.49 (0.60 to 12.37) | 0.213 |

Odds ratio orientation: odds of open reduction in PR ≤2 mm divided by those in PR >2 mm. Exact conditional confidence intervals are wide.

### Overall

## Supplementary Table S6 Recorded rehabilitation variables by reduction group

| **Field (weeks)** | **AR** | **PR** | **NR** | **Raw P** | **Holm P** |
| --- | --- | --- | --- | --- | --- |
| Non-weightbearing record | 4.91 ± 0.57 | 5.10 ± 0.97 | 5.21 ± 0.50 | 0.214 | 0.214 |
| Bilateral-crutch duration | 1.22 ± 0.11 | 1.30 ± 0.19 | 1.80 ± 0.10 | <0.001 | <0.001 |
| Single-crutch record | 3.11 ± 0.57 | 3.25 ± 0.52 | 2.91 ± 0.50 | 0.005 | 0.016 |
| Full-weightbearing record | 10.32 ± 1.26 | 9.76 ± 1.61 | 9.40 ± 1.00 | 0.011 | 0.021 |

Mean ± SD, P values use Kruskal-Wallis with Holm correction across four fields within each population.

### Garden I-III

| **Field (weeks)** | **AR** | **PR-2** | **PR-4** | **PR-6** | **NR** | **Raw P** | **Holm P** |
| --- | --- | --- | --- | --- | --- | --- | --- |
| Non-weightbearing record | 4.89 ± 0.55 | 4.62 ± 0.83 | 5.53 ± 1.08 | 5.57 ± 0.89 | 5.21 ± 0.52 | <0.001 | <0.001 |
| Bilateral-crutch duration | 1.22 ± 0.11 | 1.16 ± 0.08 | 1.43 ± 0.11 | 1.07 ± 0.09 | 1.80 ± 0.10 | <0.001 | <0.001 |
| Single-crutch record | 3.09 ± 0.55 | 3.11 ± 0.41 | 3.16 ± 0.54 | 3.83 ± 0.44 | 2.91 ± 0.52 | 0.002 | 0.003 |
| Full-weightbearing record | 10.38 ± 1.35 | 9.98 ± 1.60 | 9.61 ± 1.79 | 9.23 ± 1.19 | 9.56 ± 1.06 | 0.131 | 0.131 |

### All-Garden 5 groups

| **Field (weeks)** | **AR** | **PR-2** | **PR-4** | **PR-6** | **NR** | **Raw P** | **Holm P** |
| --- | --- | --- | --- | --- | --- | --- | --- |
| Non-weightbearing record | 4.91 ± 0.57 | 4.63 ± 0.78 | 5.53 ± 1.02 | 5.41 ± 0.80 | 5.21 ± 0.50 | <0.001 | <0.001 |
| Bilateral-crutch duration | 1.22 ± 0.11 | 1.20 ± 0.12 | 1.45 ± 0.12 | 1.22 ± 0.24 | 1.80 ± 0.10 | <0.001 | <0.001 |
| Single-crutch record | 3.11 ± 0.57 | 3.10 ± 0.40 | 3.25 ± 0.57 | 3.69 ± 0.50 | 2.91 ± 0.50 | <0.001 | <0.001 |
| Full-weightbearing record | 10.32 ± 1.26 | 10.02 ± 1.57 | 9.51 ± 1.70 | 9.62 ± 1.49 | 9.40 ± 1.00 | 0.016 | 0.016 |

## Supplementary Table S7 Rehabilitation records and functional outcomes

| **Rehabilitation field** | **Outcome** | **Spearman rho** | **Holm P** |
| --- | --- | --- | --- |
| Non-weightbearing record | 6-month HHS | 0.682 | <0.001 |
| Non-weightbearing record | 12-month HHS | -0.032 | 0.666 |
| Non-weightbearing record | Paired 6-to-12-month change | -0.627 | <0.001 |
| Bilateral-crutch duration | 6-month HHS | 0.283 | <0.001 |
| Bilateral-crutch duration | 12-month HHS | -0.413 | <0.001 |
| Bilateral-crutch duration | Paired 6-to-12-month change | -0.652 | <0.001 |
| Single-crutch record | 6-month HHS | 0.670 | <0.001 |
| Single-crutch record | 12-month HHS | 0.282 | <0.001 |
| Single-crutch record | Paired 6-to-12-month change | -0.308 | <0.001 |
| Full-weightbearing record | 6-month HHS | -0.370 | <0.001 |
| Full-weightbearing record | 12-month HHS | 0.109 | 0.290 |
| Full-weightbearing record | Paired 6-to-12-month change | 0.411 | <0.001 |

Overall cohort n=180.

### Garden I-III cohort

| **Rehabilitation field** | **Outcome** | **Spearman rho** | **Holm P** |
| --- | --- | --- | --- |
| Non-weightbearing record | 6-month HHS | 0.694 | <0.001 |
| Non-weightbearing record | 12-month HHS | -0.123 | 0.413 |
| Non-weightbearing record | Paired 6-to-12-month change | -0.706 | <0.001 |
| Bilateral-crutch duration | 6-month HHS | 0.347 | <0.001 |
| Bilateral-crutch duration | 12-month HHS | -0.354 | <0.001 |
| Bilateral-crutch duration | Paired 6-to-12-month change | -0.607 | <0.001 |
| Single-crutch record | 6-month HHS | 0.643 | <0.001 |
| Single-crutch record | 12-month HHS | 0.126 | 0.413 |
| Single-crutch record | Paired 6-to-12-month change | -0.451 | <0.001 |
| Full-weightbearing record | 6-month HHS | -0.396 | <0.001 |
| Full-weightbearing record | 12-month HHS | 0.119 | 0.413 |
| Full-weightbearing record | Paired 6-to-12-month change | 0.431 | <0.001 |

Garden I-III cohort n=140.

## Supplementary Table S8 Postoperative rehabilitation sensitivity coefficients

| **Population** | **Added field per week** | **12-month HHS coefficient (95% CI)** | **Paired change coefficient (95% CI)** |
| --- | --- | --- | --- |
| Overall | Non-weightbearing record | -1.00 (-2.82 to 0.82); P 0.279 | -10.23 (-11.94 to -8.53); P <0.001 |
| Overall | Bilateral-crutch duration | 4.34 (-6.34 to 15.03); P 0.424 | -31.88 (-46.33 to -17.43); P <0.001 |
| Overall | Single-crutch record | 1.13 (-1.54 to 3.80); P 0.405 | -13.24 (-16.29 to -10.20); P <0.001 |
| Overall | Full-weightbearing record | -0.12 (-1.02 to 0.77); P 0.789 | 3.21 (2.10 to 4.32); P <0.001 |
| Garden I-III | Non-weightbearing record | 1.11 (-0.37 to 2.60); P 0.140 | -8.89 (-10.37 to -7.40); P <0.001 |
| Garden I-III | Bilateral-crutch duration | 16.38 (5.17 to 27.59); P 0.005 | -62.83 (-78.40 to -47.26); P <0.001 |
| Garden I-III | Single-crutch record | 3.28 (1.03 to 5.52); P 0.005 | -12.57 (-15.68 to -9.45); P <0.001 |
| Garden I-III | Full-weightbearing record | -0.51 (-1.33 to 0.31); P 0.224 | 3.13 (2.23 to 4.03); P <0.001 |
| All-Garden 5 groups | Non-weightbearing record | 0.94 (-0.58 to 2.45); P 0.223 | -8.87 (-10.34 to -7.40); P <0.001 |
| All-Garden 5 groups | Bilateral-crutch duration | 20.93 (12.21 to 29.64); P <0.001 | -33.40 (-48.64 to -18.16); P <0.001 |
| All-Garden 5 groups | Single-crutch record | 3.50 (1.40 to 5.61); P 0.001 | -11.14 (-13.74 to -8.54); P <0.001 |
| All-Garden 5 groups | Full-weightbearing record | -0.40 (-1.22 to 0.41); P 0.328 | 2.80 (1.92 to 3.68); P <0.001 |

Each row represents two separate expanded models with one rehabilitation field added.

### Overall 3 groups group contrasts after rehabilitation adjustment

| **Added field** | **n** | **Comparison** | **HHS difference (95% CI)** | **P** |
| --- | --- | --- | --- | --- |
| Non-weightbearing record | 180 | NR vs AR | -24.92 (-27.10 to -22.75) | <0.001 |
| Non-weightbearing record | 180 | PR vs AR | -9.24 (-11.26 to -7.23) | <0.001 |
| Bilateral-crutch duration | 180 | NR vs AR | -27.82 (-34.13 to -21.52) | <0.001 |
| Bilateral-crutch duration | 180 | PR vs AR | -9.97 (-12.29 to -7.65) | <0.001 |
| Single-crutch record | 180 | NR vs AR | -25.20 (-27.12 to -23.29) | <0.001 |
| Single-crutch record | 180 | PR vs AR | -9.82 (-11.69 to -7.95) | <0.001 |
| Full-weightbearing record | 180 | NR vs AR | -25.47 (-27.50 to -23.44) | <0.001 |
| Full-weightbearing record | 180 | PR vs AR | -9.64 (-11.68 to -7.60) | <0.001 |

### Garden I-III 5 groups group contrasts after rehabilitation adjustment

| **Added field** | **n** | **Comparison** | **HHS difference (95% CI)** | **P** |
| --- | --- | --- | --- | --- |
| Non-weightbearing record | 140 | NR vs AR | -25.19 (-27.41 to -22.96) | <0.001 |
| Non-weightbearing record | 140 | PR-2 vs AR | -4.18 (-6.07 to -2.29) | <0.001 |
| Non-weightbearing record | 140 | PR-4 vs AR | -15.05 (-17.25 to -12.85) | <0.001 |
| Non-weightbearing record | 140 | PR-6 vs AR | -23.13 (-27.52 to -18.73) | <0.001 |
| Bilateral-crutch duration | 140 | NR vs AR | -34.45 (-40.93 to -27.97) | <0.001 |
| Bilateral-crutch duration | 140 | PR-2 vs AR | -3.68 (-5.49 to -1.87) | <0.001 |
| Bilateral-crutch duration | 140 | PR-4 vs AR | -18.07 (-20.82 to -15.31) | <0.001 |
| Bilateral-crutch duration | 140 | PR-6 vs AR | -20.00 (-24.22 to -15.78) | <0.001 |
| Single-crutch record | 140 | NR vs AR | -24.30 (-26.18 to -22.41) | <0.001 |
| Single-crutch record | 140 | PR-2 vs AR | -4.66 (-6.42 to -2.91) | <0.001 |
| Single-crutch record | 140 | PR-4 vs AR | -14.79 (-17.09 to -12.49) | <0.001 |
| Single-crutch record | 140 | PR-6 vs AR | -24.92 (-29.28 to -20.55) | <0.001 |
| Full-weightbearing record | 140 | NR vs AR | -25.13 (-27.45 to -22.81) | <0.001 |
| Full-weightbearing record | 140 | PR-2 vs AR | -4.65 (-6.63 to -2.67) | <0.001 |
| Full-weightbearing record | 140 | PR-4 vs AR | -14.66 (-16.94 to -12.38) | <0.001 |
| Full-weightbearing record | 140 | PR-6 vs AR | -22.90 (-27.49 to -18.31) | <0.001 |

## Supplementary Table S9 Paired HHS change and longitudinal interaction

| **Population** | **Group** | **n 6-12** | **Paired 6-12 change median (IQR)** | **n 12-18** | **Paired 12-18 change median (IQR)** |
| --- | --- | --- | --- | --- | --- |
| Overall | AR | 45 | 10.00 (10.00 to 10.00) | 42 | -2.00 (-2.00 to -1.24) |
| Overall | PR | 98 | 11.85 (5.17 to 21.68) | 93 | -0.33 (-5.98 to 7.86) |
| Overall | NR | 37 | -6.00 (-6.00 to -6.00) | 36 | 5.00 (5.00 to 5.00) |
| Garden I-III | AR | 38 | 10.00 (10.00 to 10.00) | 36 | -2.00 (-2.00 to -2.00) |
| Garden I-III | PR-2 | 39 | 22.10 (16.18 to 27.01) | 35 | -0.65 (-5.38 to 4.63) |
| Garden I-III | PR-4 | 33 | 5.98 (-4.81 to 15.89) | 33 | -2.44 (-10.92 to 6.10) |
| Garden I-III | PR-6 | 10 | -2.27 (-11.09 to 4.17) | 10 | 0.49 (-3.03 to 8.61) |
| Garden I-III | NR | 20 | -6.00 (-6.00 to -6.00) | 19 | 5.00 (5.00 to 5.00) |

| **Population** | **Months** | **Patients** | **Records** | **Interaction F (df1, df2)** | **P** |
| --- | --- | --- | --- | --- | --- |
| Overall three groups | 6/12 | 180 | 360 | 1828.06 (2, 179) | <0.001 |
| Overall three groups | 6/12/18 | 180 | 531 | 927.03 (4, 179) | <0.001 |
| Garden I-III five groups | 6/12 | 140 | 280 | 950.56 (4, 139) | <0.001 |
| Garden I-III five groups | 6/12/18 | 140 | 413 | 609.86 (8, 139) | <0.001 |
| All-Garden five groups | 6/12 | 180 | 360 | 956.56 (4, 179) | <0.001 |
| All-Garden five groups | 6/12/18 | 180 | 531 | 647.07 (8, 179) | <0.001 |

Records denotes the number of available HHS observations across the indicated visits. Patient-clustered group-by-time interaction models adjusted for age, sex, and Garden classification.

## Supplementary Table S10 Recomputed previously described sensitivity analyses

| **Model** | **n** | **Comparison** | **HHS difference (95% CI)** | **P** |
| --- | --- | --- | --- | --- |
| Overall HC3 | 180 | NR vs AR | -25.62 (-27.42 to -23.82) | <0.001 |
| Overall HC3 | 180 | PR vs AR | -9.40 (-11.34 to -7.47) | <0.001 |
| Overall median quantile | 180 | NR vs AR | -25.50 (-27.96 to -23.05) | <0.001 |
| Overall median quantile | 180 | PR vs AR | -7.99 (-9.94 to -6.05) | <0.001 |
| Garden I-III median quantile | 140 | NR vs AR | -26.60 (-29.33 to -23.86) | <0.001 |
| Garden I-III median quantile | 140 | PR-2 vs AR | -5.14 (-7.39 to -2.89) | <0.001 |
| Garden I-III median quantile | 140 | PR-4 vs AR | -14.07 (-16.44 to -11.70) | <0.001 |
| Garden I-III median quantile | 140 | PR-6 vs AR | -22.01 (-25.48 to -18.55) | <0.001 |
| Garden II-III HC3 | 127 | NR vs AR | -25.12 (-27.71 to -22.53) | <0.001 |
| Garden II-III HC3 | 127 | PR vs AR | -10.83 (-13.10 to -8.56) | <0.001 |
| Overall overlap-weighted | 180 | NR vs AR | -24.48 (-27.11 to -21.85) | <0.001 |
| Overall overlap-weighted | 180 | PR vs AR | -8.89 (-11.30 to -6.49) | <0.001 |
| Garden I-III ordinal | 140 | Per category | -6.72 (-7.29 to -6.14) | <0.001 |

All analyses use corrected ages. The five-level ordinal score orders AR, PR-2, PR-4, PR-6, NR.

**Supplementary Table S11 Primary surgeon case counts**

| **Primary surgeon code** | **AR, n** | **PR, n** | **NR, n** | **Total, n** |
| --- | --- | --- | --- | --- |
| A1 | 3 | 4 | 3 | 10 |
| A2 | 2 | 8 | 3 | 13 |
| A3 | 2 | 5 | 2 | 9 |
| A4 | 5 | 4 | 3 | 12 |
| A5 | 2 | 5 | 0 | 7 |
| B1 | 4 | 4 | 1 | 9 |
| B2 | 0 | 8 | 0 | 8 |
| B3 | 3 | 7 | 1 | 11 |
| B4 | 0 | 3 | 2 | 5 |
| B5 | 3 | 3 | 8 | 14 |
| C1 | 0 | 5 | 2 | 7 |
| C2 | 3 | 6 | 0 | 9 |
| C3 | 2 | 4 | 0 | 6 |
| D1 | 2 | 1 | 2 | 5 |
| D2 | 0 | 3 | 0 | 3 |
| D3 | 2 | 4 | 1 | 7 |
| E1 | 0 | 4 | 2 | 6 |
| E2 | 1 | 0 | 1 | 2 |
| E3 | 2 | 2 | 2 | 6 |
| F1 | 1 | 6 | 3 | 10 |
| F2 | 1 | 1 | 1 | 3 |
| G1 | 6 | 6 | 0 | 12 |
| G2 | 1 | 5 | 0 | 6 |

The unchanged case-count table previously presented as the continuation of main Table 1. The main table retains the surgeon-count summary, and surgeon sensitivity models remain in Supplementary Table S4.

**Supplementary Table S12 Age restriction below 60 years**

**S12A Cohort composition**

| **Population** | **Group** | **All ages n** | **Age <60 n** | **Excluded age ≥60 n** |
| --- | --- | --- | --- | --- |
| Overall three groups | All | 180 | 160 | 20 |
| Overall three groups | AR | 45 | 42 | 3 |
| Overall three groups | PR | 98 | 82 | 16 |
| Overall three groups | NR | 37 | 36 | 1 |
| Garden I-III five groups | All | 140 | 121 | 19 |
| Garden I-III five groups | AR | 38 | 36 | 2 |
| Garden I-III five groups | PR-2 | 39 | 31 | 8 |
| Garden I-III five groups | PR-4 | 33 | 27 | 6 |
| Garden I-III five groups | PR-6 | 10 | 8 | 2 |
| Garden I-III five groups | NR | 20 | 19 | 1 |
| All-Garden five groups | All | 180 | 160 | 20 |
| All-Garden five groups | AR | 45 | 42 | 3 |
| All-Garden five groups | PR-2 | 45 | 37 | 8 |
| All-Garden five groups | PR-4 | 38 | 32 | 6 |
| All-Garden five groups | PR-6 | 15 | 13 | 2 |
| All-Garden five groups | NR | 37 | 36 | 1 |

Age-unrestricted cohort: 180 patients, mean ± SD 41.6 ± 14.1 years, range 18-70. Age <60 cohort: 160 patients, 38.5 ± 11.7 years, range 18-59. The 20 excluded patients were aged 61-70; there was no recorded age of exactly 60.

**S12B Overall three-group comparison (n = 180 versus 160)**

| **Model** | **Comparison** | **All ages HHS difference (95% CI)** | **P** | **Age <60 HHS difference (95% CI)** | **P** |
| --- | --- | --- | --- | --- | --- |
| Base | PR vs AR | -9.40 (-11.34 to -7.47) | <0.001 | -9.70 (-11.80 to -7.61) | <0.001 |
| Base | NR vs AR | -25.62 (-27.42 to -23.82) | <0.001 | -25.78 (-27.67 to -23.89) | <0.001 |
| Expanded | PR vs AR | -9.55 (-11.54 to -7.56) | <0.001 | -9.96 (-12.12 to -7.80) | <0.001 |
| Expanded | NR vs AR | -25.36 (-27.32 to -23.40) | <0.001 | -25.43 (-27.52 to -23.35) | <0.001 |

Base adjusts for continuous age, sex, and categorical Garden classification. Expanded additionally adjusts for open reduction, injury-to-surgery interval, Pauwels angle, posterior tilt, BMI, and smoking. Both age columns use HC3 covariance and two-sided t-based inference with residual degrees of freedom, including the base model. These base-model intervals therefore differ from the conventional intervals retained in main Tables 4 and 5, although their point estimates are identical. AR is the reference.

**S12C Garden I-III five-group comparison (n = 140 versus 121)**

| **Model** | **Comparison** | **All ages HHS difference (95% CI)** | **P** | **Age <60 HHS difference (95% CI)** | **P** |
| --- | --- | --- | --- | --- | --- |
| Base | PR-2 vs AR | -4.54 (-6.40 to -2.68) | <0.001 | -5.19 (-7.31 to -3.08) | <0.001 |
| Base | PR-4 vs AR | -14.25 (-16.48 to -12.03) | <0.001 | -14.88 (-17.02 to -12.73) | <0.001 |
| Base | PR-6 vs AR | -22.60 (-26.97 to -18.22) | <0.001 | -22.66 (-27.36 to -17.96) | <0.001 |
| Base | NR vs AR | -24.92 (-27.11 to -22.74) | <0.001 | -25.03 (-27.21 to -22.85) | <0.001 |
| Expanded | PR-2 vs AR | -4.36 (-6.29 to -2.44) | <0.001 | -5.06 (-7.35 to -2.78) | <0.001 |
| Expanded | PR-4 vs AR | -14.18 (-16.60 to -11.77) | <0.001 | -14.81 (-17.23 to -12.40) | <0.001 |
| Expanded | PR-6 vs AR | -22.29 (-26.73 to -17.85) | <0.001 | -22.28 (-27.13 to -17.44) | <0.001 |
| Expanded | NR vs AR | -24.73 (-27.17 to -22.29) | <0.001 | -24.81 (-27.29 to -22.32) | <0.001 |

Base adjusts for continuous age, sex, and categorical Garden classification. Expanded additionally adjusts for open reduction, injury-to-surgery interval, Pauwels angle, posterior tilt, BMI, and smoking. Both age columns use HC3 covariance and two-sided t-based inference with residual degrees of freedom.

**S12D All-Garden five-group comparison (n = 180 versus 160)**

| **Model** | **Comparison** | **All ages HHS difference (95% CI)** | **P** | **Age <60 HHS difference (95% CI)** | **P** |
| --- | --- | --- | --- | --- | --- |
| Base | PR-2 vs AR | -4.49 (-6.34 to -2.64) | <0.001 | -5.04 (-7.11 to -2.96) | <0.001 |
| Base | PR-4 vs AR | -12.16 (-14.47 to -9.84) | <0.001 | -12.38 (-14.75 to -10.00) | <0.001 |
| Base | PR-6 vs AR | -16.83 (-21.96 to -11.70) | <0.001 | -16.03 (-21.61 to -10.46) | <0.001 |
| Base | NR vs AR | -25.87 (-27.79 to -23.95) | <0.001 | -25.95 (-27.94 to -23.96) | <0.001 |
| Expanded | PR-2 vs AR | -4.62 (-6.47 to -2.78) | <0.001 | -5.36 (-7.39 to -3.33) | <0.001 |
| Expanded | PR-4 vs AR | -12.36 (-14.76 to -9.96) | <0.001 | -12.71 (-15.14 to -10.28) | <0.001 |
| Expanded | PR-6 vs AR | -16.69 (-21.86 to -11.52) | <0.001 | -16.05 (-21.66 to -10.43) | <0.001 |
| Expanded | NR vs AR | -25.46 (-27.48 to -23.44) | <0.001 | -25.51 (-27.62 to -23.39) | <0.001 |

Base adjusts for continuous age, sex, and categorical Garden classification. Expanded additionally adjusts for open reduction, injury-to-surgery interval, Pauwels angle, posterior tilt, BMI, and smoking. Both age columns use HC3 covariance and two-sided t-based inference with residual degrees of freedom.

**S12E Continuous displacement within PR**

**All-Garden PR (n = 98 versus 82)**

| **Model** | **Comparison** | **All ages HHS difference (95% CI)** | **P** | **Age <60 HHS difference (95% CI)** | **P** |
| --- | --- | --- | --- | --- | --- |
| Base | Per mm | -2.93 (-3.78 to -2.07) | <0.001 | -2.65 (-3.58 to -1.72) | <0.001 |
| Expanded | Per mm | -2.82 (-3.75 to -1.88) | <0.001 | -2.55 (-3.58 to -1.52) | <0.001 |

**Garden I-III PR (n = 82 versus 66)**

| **Model** | **Comparison** | **All ages HHS difference (95% CI)** | **P** | **Age <60 HHS difference (95% CI)** | **P** |
| --- | --- | --- | --- | --- | --- |
| Base | Per mm | -3.89 (-4.66 to -3.12) | <0.001 | -3.77 (-4.67 to -2.87) | <0.001 |
| Expanded | Per mm | -3.89 (-4.66 to -3.11) | <0.001 | -3.70 (-4.65 to -2.75) | <0.001 |

Effects are HHS points per additional millimeter. PR cases only are included; AR was not assigned zero displacement.

## Model diagnostics and completeness

| **Model** | **n** | **Rank/columns** | **R²** | **Shapiro P** | **BP P** |
| --- | --- | --- | --- | --- | --- |
| overall_3_base_HC3 | 180 | 8/8 | 0.678 | <0.001 | <0.001 |
| restricted_5_base_HC3 | 140 | 9/9 | 0.816 | 0.196 | 0.162 |
| fullgarden_5_base_HC3 | 180 | 10/10 | 0.781 | 0.009 | <0.001 |
| overall_3_expanded | 180 | 15/15 | 0.697 | <0.001 | 0.011 |
| restricted_5_expanded | 140 | 16/16 | 0.825 | 0.120 | 0.377 |
| fullgarden_5_expanded | 180 | 17/17 | 0.794 | 0.024 | <0.001 |
| PR_all_continuous | 98 | 7/7 | 0.474 | 0.099 | 0.166 |
| PR_restricted_continuous | 82 | 6/6 | 0.593 | 0.465 | 0.418 |

BP denotes the Breusch-Pagan test. Overall_3 denotes the overall Garden I-IV three-group cohort; restricted_5, the Garden I-III five-group cohort; fullgarden_5, the all-Garden five-group cohort. PR_all and PR_restricted denote PR-only analyses in the corresponding Garden I-IV and Garden I-III populations. Base models adjust for age, sex, and categorical Garden classification; expanded models add the clinical covariates defined above; continuous denotes displacement per millimeter. HC3 denotes heteroscedasticity-consistent covariance.

# Supplementary Figures

## Supplementary Figure S1

Supplementary Figure S1. Individual Harris Hip Score (HHS) distributions in the Garden I–IV cohort. Panels a, b and c show the recorded 6-, 12- and 18-month assessments, respectively. Each small symbol denotes one participant at that assessment. Large horizontal bars mark group medians; vertical bars span the 25th–75th percentiles (interquartile range, IQR). Sample sizes appear beneath group labels. Missing 18-month records are omitted from that assessment only. AR, anatomical reduction; PR, positive-buttress reduction; NR, negative-buttress reduction. The figure is descriptive and does not encode significance tests.

## Supplementary Figure S2

Supplementary Figure S2. Individual Harris Hip Score (HHS) distributions in the Garden I–III cohort. Panels a, b and c show the recorded 6-, 12- and 18-month assessments, respectively. Each small symbol denotes one participant at that assessment. Large horizontal bars mark group medians; vertical bars span the 25th–75th percentiles (interquartile range, IQR). Sample sizes appear beneath group labels. Missing 18-month records are omitted from that assessment only. AR, anatomical reduction; PR, positive-buttress reduction; NR, negative-buttress reduction. PR-2, ≤2 mm; PR-4, >2–4 mm; PR-6, >4 mm residual displacement. The figure is descriptive and does not encode significance tests.

## Supplementary Figure S3

Supplementary Figure S3. Continuous residual positive displacement and 12-month Harris Hip Score (HHS). Panel a includes all 98 participants with positive-buttress reduction (PR), including Garden IV fractures; panel b includes the 82 PR participants with Garden I–III fractures. Gray points are individual observed HHS values without covariate adjustment. Red lines are linear-model estimated marginal means standardized to the age, sex and categorical Garden distribution of each respective PR cohort. Shaded bands are two-sided 95% confidence intervals for these standardized means, using HC3 covariance and Student's t distribution with residual degrees of freedom. The models estimate an association per millimetre and do not identify or validate a clinical threshold. All eligible PR observations are retained.

## Supplementary Figure S4

Supplementary Figure S4. Five-group 12-month Harris Hip Score (HHS) sensitivity analysis retaining Garden IV fractures (n = 180). Points show adjusted differences relative to anatomical reduction (AR); horizontal bars are two-sided 95% confidence intervals. Black circles show the base model adjusting for age, sex and categorical Garden classification. Gray squares additionally adjust for open reduction, injury-to-surgery interval, Pauwels angle, posterior tilt, body mass index and smoking category. These two models use HC3 covariance and Student's t distribution with residual degrees of freedom. Red diamonds show the same expanded point estimates with surgeon-clustered CR1 covariance, small-sample correction and t-based inference over 22 degrees of freedom (23 surgeon codes). Clustering addresses correlated residuals, not surgeon-level confounding by itself. PR-2, residual positive displacement ≤2 mm; PR-4, >2–4 mm; PR-6, >4 mm; NR, negative-buttress reduction.

## Supplementary Figure S5

Supplementary Figure S5. Open-reduction proportions across the five reduction groups. Panel a includes all 180 participants (Garden I–IV); panel b includes 140 participants after exclusion of Garden IV fractures. Bar heights are observed percentages, and labels show the number undergoing open reduction divided by the group denominator. Global two-sided Fisher–Freeman–Halton exact tests compare the five groups separately within each cohort. AR, anatomical reduction; PR-2, positive-buttress displacement ≤2 mm; PR-4, >2–4 mm; PR-6, >4 mm; NR, negative-buttress reduction.
